# Quadruple Helix Inversions of Liquid Crystals Orchestrated by Direct Chirality Transmission of an Intrinsically Chiral Light‐Driven Molecular Machine

**DOI:** 10.1002/adma.202513156

**Published:** 2025-10-08

**Authors:** Jinyu Sheng, Alexander Ryabchun, Guiying Long, Ben L. Feringa

**Affiliations:** ^1^ Stratingh Institute for Chemistry University of Groningen Nijenborgh 3 Groningen 9747 AG the Netherlands; ^2^ College of Chemistry Chemical Engineering and Materials Science Soochow University Suzhou Jiangsu 215123 China

**Keywords:** chirality, chirality inversion, light‐responsive materials, liquid crystals, molecular motors, NIR light

## Abstract

Endowing liquid crystal (LC) soft matter with stimuli‐responsiveness is pivotal for programmable and dynamic photonics in the next generation of optical materials. However, suitable molecular candidates with intrinsic dynamic chirality that can twist LC helices using external stimuli in a multistate, distinct manner are still very rare. In this study, an elaborately designed intrinsically chiral molecular machine is presented with four‐state dynamic chirality and mesogenic units. This design enables direct chirality transmission, leading to sequential quadruple helix inversions of the LC architecture via unidirectional rotary motion, achieving a high helical twisting power in all states. The inversion of the supramolecular helicity by near‐infrared (NIR) light is demonstrated by NIR light‐triggered isomerization of the molecular motor structure through an efficient radiative energy transfer pathway from upconversion nanoparticles in the LC film. Hence, these results demonstrate a unique photoresponsive LC system with high robustness, NIR light triggering, and multistate helix tunability with a fascinating potential for constructing smart optical devices.

## Graphical abstract

A molecular motor with four‐state dynamic chirality is integrated into liquid crystals, enabling quadruple sequential helix inversions and high helical twisting power. Due to high motor efficiency, the supramolecular helicity can be controlled by near‐infrared light via upconversion nanoparticles, resulting in a robust, multistate, photoresponsive soft matter. This concept provides new opportunities for programmable photonic smart materials.

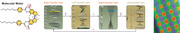

## 1 Introduction

In nature, dynamic chirality transmission across different length scales plays an important role in the emergence of life. Homochirality is widely considered as a biosignature,^[^ ^1^ ^]^ and understanding the correlation between small chiral molecules and biological functions inspires scientists to design macroscopic artificial systems capable of performing life‐like motions and, ultimately, complex dynamic functions.^[^ ^2^ ^]^ However, the intricacy of the responsive behavior of these artificial systems lags far behind that of natural biological systems. These observations are understandable, given that the transmission of chirality governing macroscopic motion needs essential parameters such as a hierarchical molecular organization along length scales, cooperativity, and synchronization of molecules.^[^ ^3^, ^4^ ^]^ Moreover, inducing macroscopic motion in functional artificial systems through external stimuli, such as light, with high temporal and spatial precision in a non‐invasive manner, is highly desirable for advancing responsive materials. Hence, developing approaches toward smart material applications by the use of dynamically chiral small molecules featuring stimuli‐responsiveness is extremely promising, yet still highly challenging.^[^ ^5^, ^6^, ^7^, ^8^, ^9^, ^10^ ^]^

**Figure 1:**
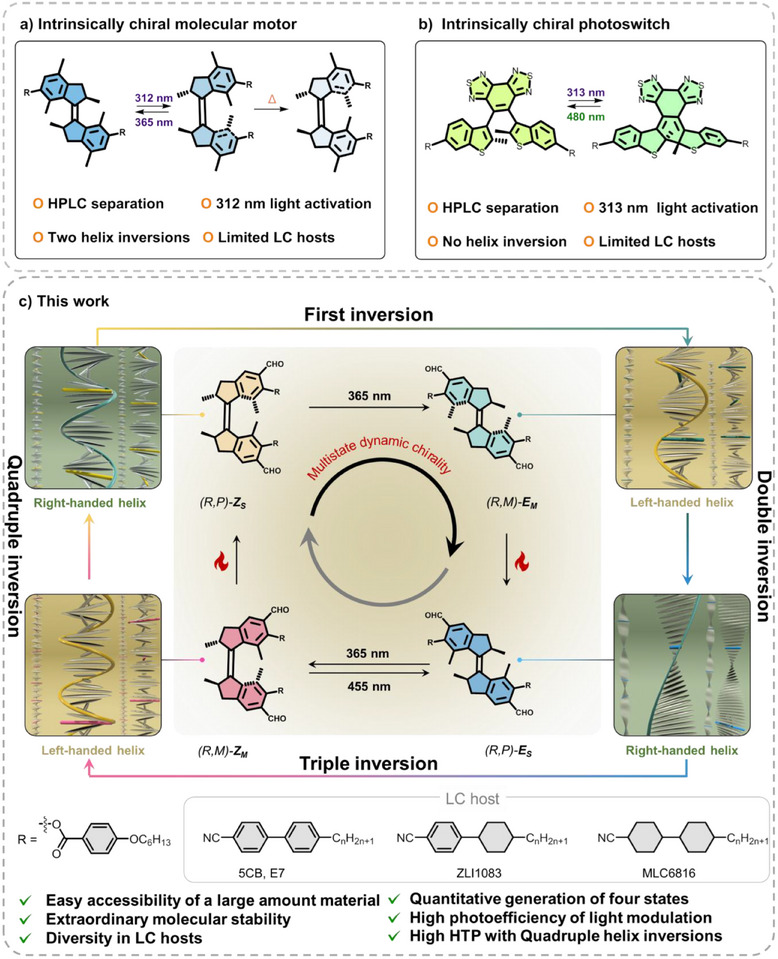
a,b) Light‐responsive LC systems based on molecular motors^[^ ^31^ ^]^ and diarylethenes.^[^ ^32^ ^]^ c) Four‐state rotary cycle of a highly photoefficient “first‐generation” molecular motor, controlling the multistate handedness of a LC with four times helicity inversion. Only one enantiomer is shown. Chemical structures of low molar mass LCs are given at the bottom.

Liquid crystals (LCs) are superior highly‐ordered soft matter with a wide range of applications in physics, chemistry, biology, optics, and material sciences.^[^ ^11^, ^12^, ^13^, ^14^ ^]^ The sensitivity of their supramolecular architecture to external stimuli is one of the main features that make them unique and useful in society, with one of their most prominent applications seen in modern displays. Chiral LCs with periodic helical superstructures are extremely appealing in technological applications due to their unique optical properties, which can be tuned by various external stimuli.^[^ ^15^ ^]^ Using light as a stimulus in the modulation of dynamic LC systems allows high spatiotemporal accuracy and is an area of great contemporary interest. The most effective way to encode photoresponsiveness of the chiral LCs is the use of small light‐active molecules, in particular photoswitches, as (chiral) dopants, to achieve tuneability and control over the optical properties of LC materials. Azobenzenes^[^ ^16^, ^17^, ^18^, ^19^ ^]^ and diarylethenes (DAEs)^[^ ^20^, ^21^ ^]^ attached to external chiral groups have boosted photoresponsive LC systems showing their excellent stability and performance in switching between two distinct supramolecular structures.^[^ ^22^ ^]^ Recently, hydrazone switches with chiral fragments have also shown promising properties in changing LC helical twisting power (HTP) values.^[^ ^23^, ^24^, ^25^ ^]^ A significant step forward in this field was the development of intrinsically chiral molecular photoswitches^[^ ^26^, ^27^, ^28^, ^29^, ^30^ ^]^ and motors^[^ ^10^ ^]^ featuring superb asymmetric induction and chiral amplification in LC phases.^[^ ^9^, ^31^ ^]^ Chiral light‐driven molecular motors are unique due to their multistate dynamic chirality, allowing for tuning multistate LCs (**Figure** **1**a) and other supramolecular systems,^[^ ^32^, ^33^, ^34^, ^35^, ^36^, ^37^, ^38^, ^39^ ^]^ while intrinsically chiral DAE switches have also shown their practicality for photo‐programming photonic materials (Figure 1b).^[^ ^12^, ^26^, ^40^, ^41^ ^]^

Nonetheless, there are severe limitations in the use of these photoresponsive LC systems for real applications: i) one main obstacle is the material accessibility as these enantiopure compounds are usually obtained by high‐performance liquid chromatography or supercritical fluid chromatography separation, which is a costly and time‐consuming process. ii) Due to the short wavelength of the UV light that activates the photoswitches, the choice of suitable LC hosts is usually extremely limited because of the overlap in their absorbance spectra. iii) Moreover, short‐wavelength UV light can induce photodegradation and damage the system, and lead to low photoefficiency in achieving bulk isomerization of the system due to the low penetration depth of UV light. iv) The HTP changes obtained with current photoresponsive dopants are generally quite small, while very few systems can achieve the inversion of helicity in current LC materials. Alteration of large HTP values in combination with helicity inversion is a key parameter to control LC systems for the next generation of smart material applications, especially in advanced applications such as camouflage color and data encryption.^[^ ^42^, ^43^ ^]^ v) Finally, multistate modulation of chirality in a non‐invasive manner to address multiple states in LC materials in a fully reversible manner is still in its infancy. Using a single switch molecule to manipulate multiple stable states of the LC phase with distinct HTP values and inverted helicity is highly significant for unique spatiotemporal precision and dimensional control in smart systems, although it remains highly challenging. The design of a single molecular candidate capable of multistate helicity inversion of the LC helix with a high photostationary state (PSS), a highly robust switching behavior, and molecular integrity is required to advance the field. Taking advantage of our recently discovered formylation strategy for boosting light‐driven molecular machines,^[^ ^44^, ^45^, ^46^ ^]^ we anticipated that by applying a formylated light‐driven molecular motor, we could address all these current unsolved challenges with one molecular design (Figure 1c). Furthermore, due to the high quantum yield of the isomerization processes using this formylated molecular motor, we successfully manipulated LC phases with high efficiency and in a reversible manner for the first time with near‐infrared light. This was achieved by doping upconversion nanoparticles (UCNPs) in the system, allowing to trigger the molecular motor rotation with 980 nm light.

## 2 Molecular Design and Synthesis

Here, we introduce an intrinsically chiral light‐driven molecular motor as a superior multistate chiral dopant,^[^ ^47^ ^]^ and the chiral precursor can be easily obtained in large amounts by a chiral resolution process. Formyl groups conjugated to the central double bonds are not only designed to red‐shift the absorption band of the molecule but also to boost the performance of the motor candidate, in particular by achieving high photochemical quantum yields, quantitative transformation of 4 states, and a superb molecular stability. Phenyl‐alkoxy moieties are introduced by a simple esterification reaction to improve the compatibility with the LC hosts and to increase the efficiency in the chirality transfer (vide infra). Additionally, molecular crowding of the ester group next to the motor fjord region will significantly increase the thermal stability of the ***E_M_*** and ***Z_M_*** forms. This elaborate design leads to multistate stable dynamic chirality of the dopant.

**Scheme 1:**
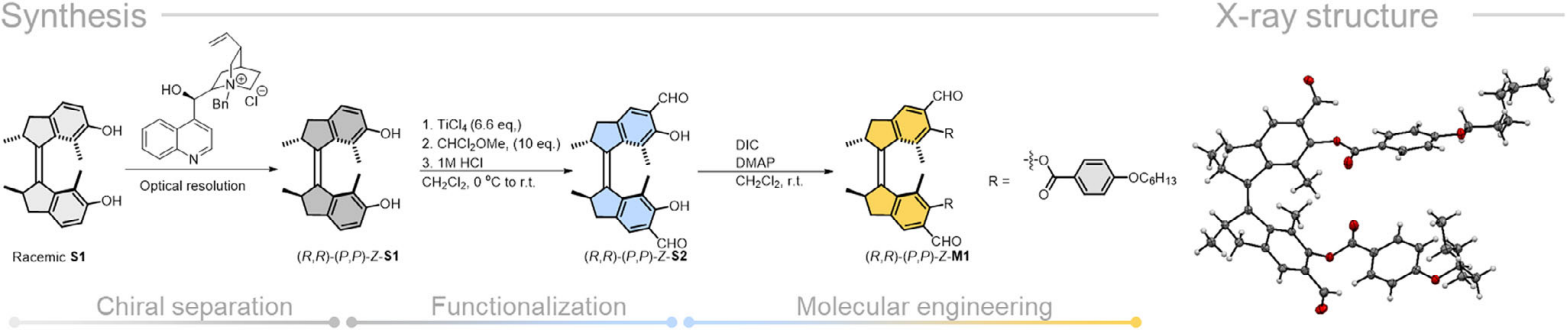
Synthetic route toward the enantiopure (*R,R*)‐(*P,P*)‐*Z*‐**M1** and its X‐ray structure.

The synthesis of **M1** includes three main steps (see **Scheme** **1**): i) Optical resolution of the racemic **S1** using N‐benzylcinchonidinium chloride for co‐crystallization to obtain chiral (*R,R*)*‐(P,P*)‐**S1** in good yield and with excellent enantiomeric excess (*e.e*., up to 98%) – a process which can be performed on a multigram scale; ii) Functionalization of (*R,R*)*‐(P,P*)**‐S1** by a simple one‐step formylation reaction to afford the high performance motor precursor (*R,R*)*‐(P,P*)‐**S2**; iii) High compatibility with the LC phase is achieved by further molecular engineering of precursor (*R,R*)*‐(P,P*)‐**S2** by a simple esterification reaction to get the final motor structure (*R,R*)*‐(P,P*)‐**M1** in high yield (90%). It is worth mentioning that the specific position for ester substitution was chosen for a higher HTP value generation in accordance with our previous studies.^[^ ^35^ ^]^ All molecules involved were fully characterized (see Figures S2–S7, Supporting information).

### 2.1 Four Distinct Chiral States: Solution Study

**Figure 2:**
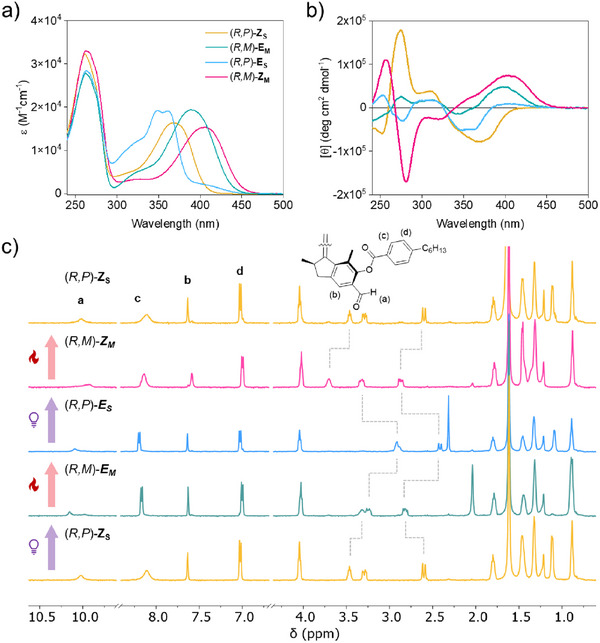
a) UV/Vis spectra (in CH_2_Cl_2_) of the four distinct isomers of motor **M1** upon isomerization, starting from (*R,P*)‐*Z*_S_. b) ECD spectra (in CH_2_Cl_2_) of the four distinct isomers of **M1** upon isomerization, starting from (*R,P*)‐*Z*_S_. c) Rotary cycle of the motor followed by ^1^H NMR spectroscopy (500 MHz, CD_2_Cl_2_) starting from (*R,P*)‐*Z*_S_ (bottom orange line) to (*R,M*)‐*E*_M_ (green line) under 365 nm light irradiation at −25 °C, followed by heating to room temperature for the THI to generate (*R,P*)‐*E*_S_ (blue line). Subsequent irradiation under 365 nm light at −25 °C resulted in the formation of (*R,M*)‐*Z*_M_ (pink line); finally, the sample was placed at room temperature to regenerate (*R,P*)‐*Z*_S_ (top orange line). All measurements were performed at −25 °C to ensure that the chemical shifts are not influenced by the temperature.

The unidirectional rotation behavior of the motor starting from (*R,R*)‐(*P,P*)‐*Z‐* **M1** (hereinafter (*R,P*)‐Z_S_) was first studied in a CH_2_Cl_2_ solution by UV–Vis, electronic circular dichroism (ECD), and proton nuclear magnetic resonance (^1^H‐NMR) spectroscopies. Irradiation of a diluted in a CH_2_Cl_2_ (*R,P*)‐Z_S_ with 365 nm light at 0 °C for 5 s led to a significant redshift of the absorption band centered at 365 to 400 nm in UV–vis spectra (**Figure** **2**a, yellow to green spectrum), indicating the formation of the (*R,R*)‐(*M,M*)‐*E‐* **M1** isomer (hereinafter (*R,M*)‐*E*_M_). Subsequently, warming the sample to ambient conditions for 10 min resulted in the absorption band shifting to 350 nm with two shoulders in the spectrum (Figure 2a, blue spectrum), in accordance with the characteristic absorption for the (*R,R*)‐(*P,P*)‐*E‐* **M1** isomer (hereinafter (*R,P*)‐*E*_S_). Continuous irradiation with 365 nm light contributed to the formation of (*R,R*)‐(*M,M*)‐*Z‐* **M1** (hereinafter (*R,M*)‐*Z*_M_), which showed an absorption band in the blue region (Figure 2a, pink spectrum). This four‐step process can also be monitored by ECD spectroscopy. Each step led to the inversion of chirality in the motor structure, resulting in dynamic Cotton effects. Interestingly, the region from 330–450 nm in the ECD spectra showed distinct changes due to the dynamic chirality, i.e., from negative to positive to negative to positive, indicating a significant difference in each chiral state, i.e., the helicity inversion (Figure 2b). ^1^H NMR spectroscopy was used to quantify the PSS of each step. Surprisingly, in situ irradiation of an NMR sample at 365 nm under ‐45 °C led to the quantitative transformation from (*R,P*)‐*Z*_S_ (Figure 2c, bottom yellow spectrum) to the (*R,M*)‐*E*_M_ isomer (Figure 2c, green spectrum), which can be further completely converted to the (*R,P*)‐*E*_S_ isomer by a thermal helix inversion (THI) step (Figure 2c, blue spectrum). Subsequent irradiation of the sample at 365 nm led to the formation of the (*R,M*)‐*Z*_M_ isomer in quantitative yield (Figure 2c, pink spectrum), which can be fully converted to the initial pure (*R,P*)‐*Z*_S_ isomer after another THI step (Figure 2c, top yellow spectra), thus completing the 4‐step unidirectional 360° rotation of the motor. As shown in Figure 2c (top spectrum), the recovered sample showed the same spectrum compared to the pristine material, proving robustness of this motor molecule. The combined results indicate that this molecule is a suitable candidate for further LC doping experiments.

## 3 Dynamic Chiral Behavior in Liquid Crystals

The cholesteric liquid crystalline phase, often termed the chiral nematic phase, is typically achieved by adding a small amount of chiral guest molecules (dopants) to a nematic LC host, causing the LC director to twist into a supramolecular helix. Chiral dopants are characterized by their HTP, which quantifies their ability to twist the nematic director, and is calculated as HTP = **p** ^−1^/**c**, where **p** is the cholesteric pitch and **c** is the concentration. The HTP is primarily determined by the chemical structure and chirality of the chiral dopant and its interaction with LC molecules. A unique optical property of the cholesteric LC materials is their ability to selectively reflect circularly polarized light, where the helical periodic supramolecular structure acts as a Bragg diffraction grating reminiscent of the “structural coloration” widely found in nature. The wavelength of the reflected light (λ_max_) is directly proportional to the helix pitch according to the equation: λ_max_ = np, where n is the average refractive index. Consequently, the color of the cholesteric material is dependent on the HTP of the chiral dopant at its constant concentration. Remote manipulation of the HTP of the chiral dopant, and, therefore the optical properties of the material with spatiotemporal control can be achieved using photoresponsive molecules.^[^ ^48^ ^]^ Over the past decade, a variety of light‐responsive chiral compounds have been developed, where molecular motors stand out due to the remarkable ability to access multiple chiral states through a single dopant with the possibility to invert chirality on demand. Chiral molecular motors have been embedded into LC media to provide tuneable optical properties,^[^ ^32^ ^]^ rotation of microscopic objects,^[^ ^33^ ^]^ macroscopic motion of soft actuators,^[^ ^49^, ^50^, ^51^ ^]^ and steering of the propulsion trajectories of microswimmers.^[^ ^52^ ^]^

First, to ascertain the efficiency of motor **M1** in twisting LCs we used Grandjean‐Cano wedge cells filled with a LC host doped with 1 wt.% of (*R,P*)‐*Z*_S_. For further simplicity, we omitted stereodescriptors since a single enantiomer was employed. More experimental details can be found in Figures S18–S22 (Supporting information). Given that HTP is contingent upon the interaction between the dopant and the LC molecules, the study was conducted for a number of commercially available LCs: i) an LC mixture containing no aromatic rings (MLC6816), ii) LCs consisting solely of aromatic biphenyl moieties (E7 and 5CB), and iii) an LC mixture containing both cyclohexyl and phenyl moieties in their structures (ZLI1083) (see Figure 1c). It is important to note that the shifted absorbance band of **M1** allowed us to use conventional cyanobiphenyl LCs, which was not possible for the unmodified first‐generation molecular motors due to a large overlap of their absorbance bands.^[^ ^35^ ^]^

**Figure 3:**
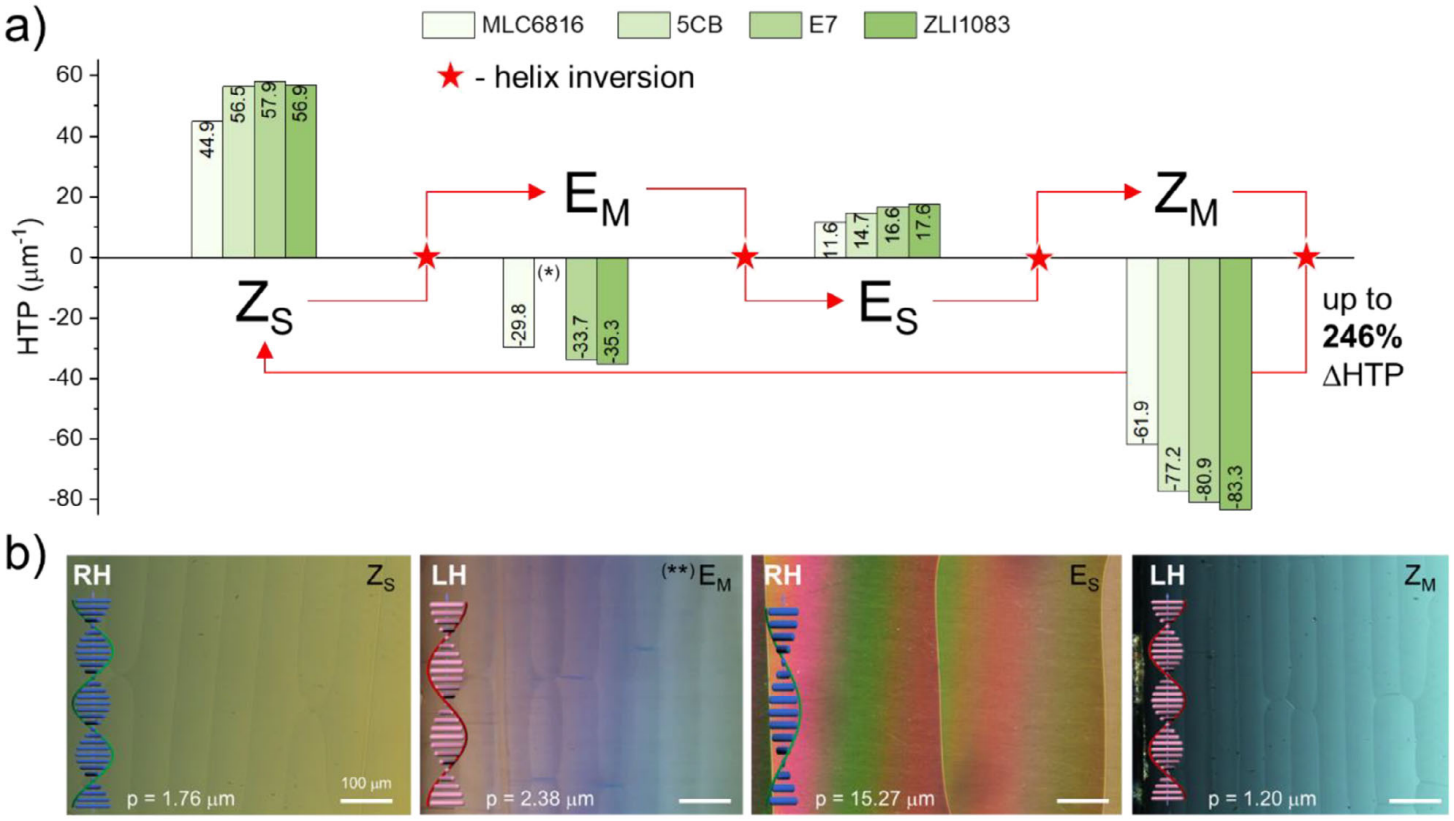
a) Helical twisting power (HTP_wt.%_ in µm^−1^) of all 4 states of the motor **M1** in different liquid crystalline media. The positive values correspond to a right‐handed cholesteric helix, while the negative values correspond to a left‐handed helix. The values were calculated based on the wt.% concentration of motor in the LC. b) Polarized optical images of Grandjean‐Cano wedge cells (*tanθ* = 0.0115) filled with different states of motor **M1** (1 wt.%) dissolved in ZLI1083. Data for *E*_S_ corresponds to *Z*_M_ irradiated with 455 nm until PSS was reached (*E*_S_:*Z*_M_ 89:11). The *E*_M_ state of the motor was prepared by irradiating *Z*_S_ with 365 nm UV light (200 mW/cm^2^) for 5 s at 5 °C. The values of the helical pitch (p) as well as the supramolecular handedness (right‐handed, (RH) and left‐handed (LH)) are provided in panel b). ^(*)^The value in 5CB cannot be determined due to crystallization of the matrix at 5 °C. ^(**)^A wedge cell with *tanθ* = 0.0192 was used. All scale bars are consistent.

The *Z*_S_ isomer of the motor induces the formation of a right‐handed supramolecular helix, which corresponds to positive values of the HTP. As can be seen in **Figure** **3**, the HTP values are rather high, exceeding those of the unsubstituted motor, and comparable to the values observed in the most advanced representatives of the first and second generations of motors.^[^ ^9^, ^35^, ^53^, ^54^ ^]^ In a non‐aromatic host (MLC6816), the motor exhibits a slightly reduced HTP value, which is likely attributed to the absence of additional π–π interactions with the host molecules. As a result of the novel molecular design, the present motor permitted the capture and study of the *E*_M_ state behavior within an LC matrix for the first time. To this end, a sample containing the initial *Z*_S_ was irradiated with 365 nm UV light for a short period (5 s) at 5 °C to significantly slow down the THI from *E*_M_ to *E*_S_. The results demonstrated that the *E*_M_ induces a left‐handed helical structure and is characterized by medium values of HTP (Table S1 and Figures S18–S22, Supporting information). Thus, UV irradiation leads to a sign inversion of the cholesteric helix mirroring the inversion of Cotton effects evidenced by the ECD data (Figure 2b). Upon heating the sample to room temperature, the THI was accelerated significantly, resulting in the formation of the *E*_S_ isomer. This process allows to produce 81–86% *E*_S_ motor. *E*_S_ can be prepared quantitatively by irradiating the solution with UV light followed by blue light. The *E*_S_ isomer exhibits positive medium HTP values in all LCs used (Figure 3a, Figure S22, and Table S1, Supporting information).

The prolonged irradiation of material containing *Z*_S_ at room temperature leads to the rapid and quantitative formation of *Z*_M_, accompanied by an inversion of the helix sign. The HTP values in all LC matrices are notably high, reaching ‐83.3 µm^−1^ in ZLI1083. As in the previous cases, the values in MLC6816 are slightly lower. The data reveal a pattern where both *Z* forms of the motor exhibit overall higher HTP values in comparison to the *E* forms. This difference can be attributed, at least in part, to the dihedral angle around C═C bond, and to the orientation of the molecular motor relative to the surrounding LC molecules. Using the racemic mixture of **M1** introduced into unidirectionally aligned LCs, this aspect was studied by means of polarized light UV–Vis spectroscopy and DFT calculations (see Figure S10, Supporting information). We show that the long molecular axes of the *Z* and *E* forms are mainly aligned parallel to the orientation of the LC molecules. Such orientation favours more efficient propagation of molecular helical chirality of the *Z* states from the motor to the LCs.^[^ ^35^, ^55^ ^]^

Notably, for the first time, we have fully characterized all states of a motor in LCs while performing the rotary cycle with four sequential helix inversions of the LC architecture. As illustrated in Figure 3 (and Table S1, Supporting information), the highest values of the variation in twisting power (≈140 µm^−1^ of absolute HTP variation, or 246%) were observed in the ZLI1083 LC material, which was employed in our further experiments.

In addition to measuring the HTPs of the molecular motor, we also conducted a comprehensive characterization of its rotational parameters in a LC medium, and compared these with the observed behavior in an organic solvent. **Table** **1** illustrates that when the motor is embedded within a crowded and ordered LC medium, the motor rotates slower by a factor of ≈3. Furthermore, the photoisomerization quantum yield of ≈50% of the motor in LCs remains nearly identical to that in solution. This is significantly higher than the quantum yield observed for any of the motors of both the first and second generations in LC systems. Thus, the enhanced photo‐efficiency, red‐shifted absorption band, elevated HTP values, and the ability to selectively target each of the four motor states that result in quadruple helix inversion of the LC superstructures offer distinct advantages for utilization in LC materials, particularly in comparison to previously reported motors (see Table S4, Supporting information).^[^ ^9^, ^22^, ^24^, ^25^, ^26^, ^35^ ^]^

**Table 1:** Gibbs activation energy (Δ^‡^G°) of the thermal helix inversion processes, half‐life times (t_1/2_) of the metastable states at 293K in solution (CH_2_Cl_2_ for the ***E*_M_** → ***E*_S_** THI step and DMSO for the ***Z*_M_** → ***Z*_S_** THI step) and in a liquid crystalline medium (ZLI1083). The quantum yields of E/Z photoisomerization were measured in acetonitrile and LC (ZLI1083), respectively.

	Solution	Liquid crystal
Δ^‡^G° (***E*_M_** → ***E*_S_**)	78.6 kJmol^−1^	81.5 kJmol^−1^
t_1/2_(***E*_M_**)	11 s	38 s
Δ^‡^G° (***Z*_M_** → ***Z*_S_**)	108.4 kJmol^−1^	111.3 kJmol^−1^
t_1/2_(***Z*_M_**)	27.3 d	90.8 d
QY(***E*_S_** → ***Z*_M_**)	51%	46%

## 4 Tuning the Optical Properties of the Cholesteric Layers

**Figure 4:**
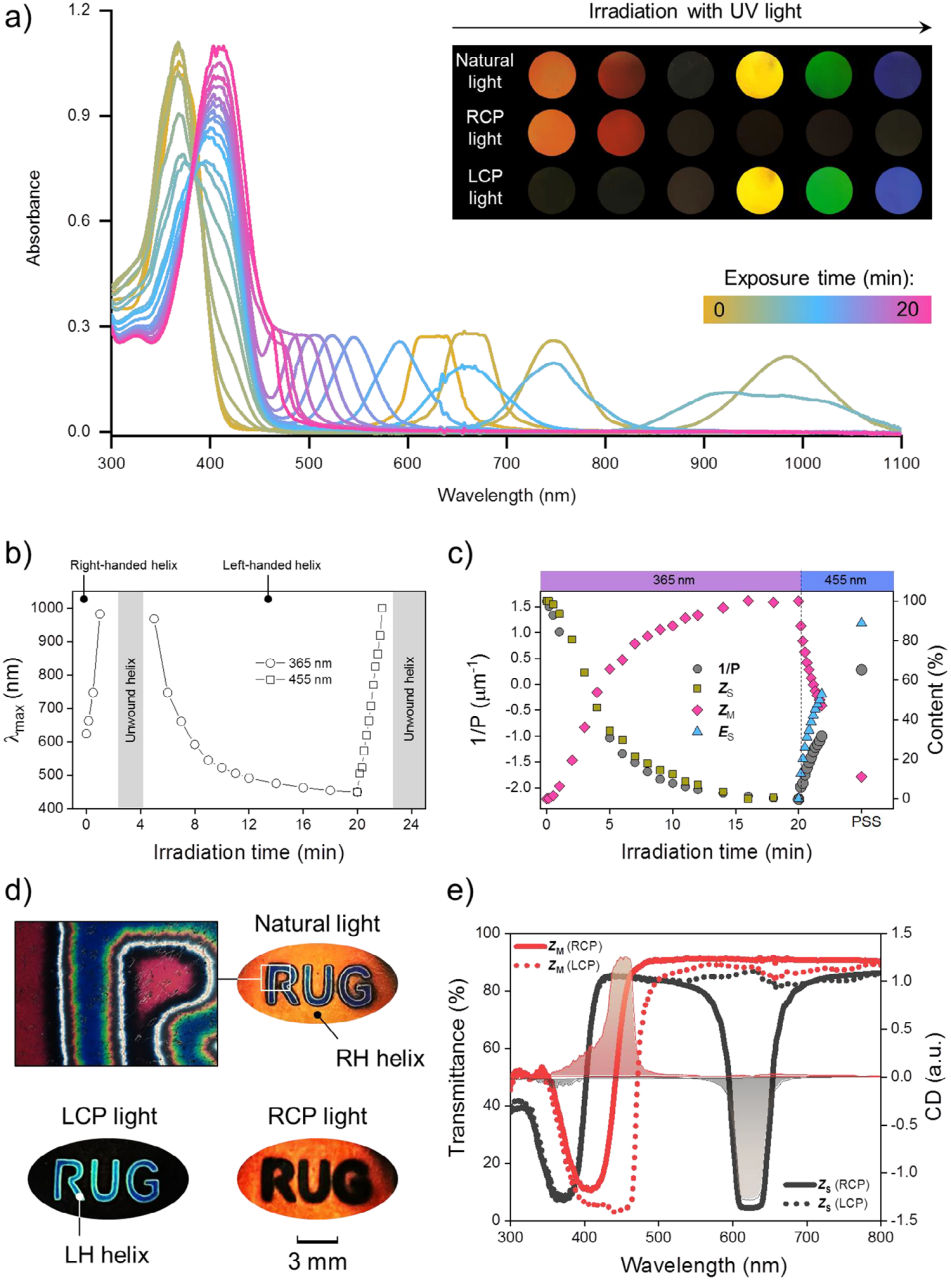
a) Change in absorbance spectra of the cell filled with a mixture of ***Z_S_*** (4.3 wt.%) in ZLI1083 upon UV light irradiation (*λ* = 365 nm). The intense absorption in the range 300–450 nm corresponds to the absorbance of the motor, while the peak on the right originates from the selective light reflection by the cholesteric supramolecular helical structure. The quartz cell had a planar boundary condition and a 6 µm gap. The intensity of the 365 nm UV light was 0.3 mWcm^−2^. The inset shows the reflection colors of the sample observed in natural (non‐polarized), right circularly polarized (RCP), and left circularly polarized (LCP) light, respectively. The black color indicates no reflection. The diameter of the circular sample is 1 cm. b) Evolution of the selective light reflection maximum (*λ*_max_) during exposure to UV light (*λ* = 365 nm) and visible light (*λ* = 455 nm, intensity 420 mWcm^−2^). c) Overlay of the kinetic profiles of the supramolecular helix pitch evolution and conversion of ***Z_S_*** to ***Z_M_*** and ***Z_M_*** to ***E_S_*** upon UV and visible light illumination, respectively. d) Real color image of the sample with a recorded pattern (abbreviation “RUG”) observed in natural, RCP, and LCP light. The pattern was recorded by UV light irradiation through the mask (365 nm, 2 min, 100 mWcm^−2^). A transmission polarized optical image is shown on the left. e) Left‐ and right‐circularly polarized (LCP and RCP) light transmission spectra (solid and dotted lines), and CD spectra (filled area) of the cholesteric layer (ZLI1083 + 4.3% (*R,P*)‐***Z_S_***) before and after exposure to UV light. The CD peaks originate from selective light reflection by the cholesteric supramolecular helices. The opposite signs of the CD signal indicate the inversion of the supramolecular chirality.

Given the rather good compatibility (up to 5 wt.%) of the motor and the LC resulting from the presence of mesogen‐like substituents, we prepared cholesteric films in a manner that enabled the selective light reflection to cover the entire visible optical range. To this end, we introduced 4.3 wt.% of ***Z*_S_** into ZLI1083. The mixture, when placed in a quartz cell with planar boundary conditions, exhibits selective reflection of right‐circularly polarized light of an orange color. Upon exposure to UV light (365 nm), the layer changes its color to red at first, after which the color disappears but then reappears, moving into the short‐wavelength region as evident from the inset in **Figure** **4**a. The absorption spectra provide clear evidence that the ***Z*_S_** state undergoes rotation to ***Z*_M_** (as indicated by the bands at 365 and 420 nm). At the same time, an additional absorption peak, resulting from light reflection by the cholesteric layer, shifts first to the infrared region of the spectrum, then vanishes entirely, and subsequently reappears, traversing the infrared and blue regions of the spectrum. In the photostationary state, the layer reflects left‐circularly polarized blue light as illustrated in Figure 4a. The observation of the sample with circular polarization filters supports the hypothesis that upon irradiation with UV light, there is an inversion of the sign of the cholesteric helicity, which is also corroborated by the circular dichroism study (Figure 4e).

The sequence of the processes can be traced on the kinetic curve of the reflection band change presented in Figure 4b. Upon irradiation with UV light, the right helical structure unwinds until it reaches the fully unwound state (or the compensated state, where the contributions of ***Z*_S_** and ***Z*_M_** compensate each other because they have opposite signs, see Table S1, Supporting information), which does not reflect light (inset to Figure 4a). Further irradiation results in the twisting of the left‐handed helical structure. The aforementioned sequence of processes can be fully reversed by irradiating the layer with blue light, which induces a photochemical transformation from ***Z*_M_** to ***E*_S_**. As we demonstrated previously, the reverse isomerization is considerably less efficient than the forward process, which essentially yields an almost quantitative conversion. The balanced design of the cholesteric mixture enabled the simultaneous observation of the motor behavior and the characteristics of the supramolecular structure. Figure 4c illustrates the correlation between the reciprocal pitch value (which is directly proportional to the HTP) and the content of each form of the motor at each time point when the sample is irradiated. The matching of the time course of these curves conveys that the optical properties of the material are entirely determined by the ratio of the chiral motor isomers that are established during motor rotation. The relatively short half‐life of ***E***_M_ in the LC medium at ambient temperature (38 s, see Table 1) poses a significant challenge for fully exploiting its properties.

Using light as a stimulus also allows to spatially control of properties as exemplified by the recording of optical patterns. Figure 4d depicts a layer of the material with a recorded color image that exhibits a distinct appearance using circularly polarized light. To record optical information, the sample was exposed to UV light for 2 min through a mask bearing the abbreviation “RUG” (Rijksuniversiteit Groningen). The irradiated area changed color and inverted chirality so that when observing the sample with left‐circularly polarized light, only the irradiated areas were visible, while the non‐irradiated areas were visible using right‐circularly polarized light. In unpolarized (natural) light, all areas of the sample were colored. The regions with opposite chirality are separated by a topological defect, which appears as a white line along the contour of the letters, clearly visible in the POM image (Figure 4d, left). On either side of this line, a gradual change in color is observed, resulting from the diffusion of both motor forms (please see Figure S23, Supporting information for more details). Additional examples of color patterns are provided in Figure S24 (Supporting information), where the patterns were erased and rewritten on the same sample. This data, together with the cycling study shown in Figure S25 (Supporting information), indicates reversibility and high fatigue resistance of the material.

Hence, we have shown that the molecular motor enables the reversible and effective tuning of the cholesteric helix, and consequently, the color of the LC material resulting from the change in the sign of chirality with spatial resolution, with the chiral handedness alternating up to four times for a full cycle of motor rotation.

## 5 Chiral States Addressed by Near Infrared Light

**Figure 5:**
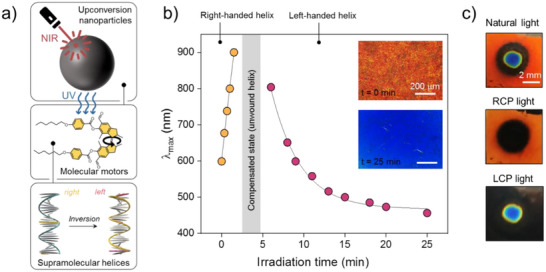
a) Schematic representation of NIR light‐induced inversion of the supramolecular helical structure facilitated by our highly efficient molecular motor. b) Evolution of the selective light reflection maximum (*λ*_max_) of the cholesteric layer (***Z*_S_** 4.5 wt.% and UCNPs 2 wt.% in ZLI1083) during exposure to NIR laser light (*λ* = 980 nm, 3.2 W). The inset shows polarized optical images of the layer before and after NIR irradiation taken in reflection mode. c) Real color image of the sample exposed to a NIR laser beam observed in natural, RCP, and LCP light. The images clearly indicate the inversion of the handedness of the supramolecular helical structures, selectively reflecting circularly polarized light.

The high efficiency of the photoisomerization steps in the motor rotary cycle allowed us to demonstrate for the first time the change in optical properties of a motor‐doped LC layer and the inversion of supramolecular chirality under the action of NIR light.^[^ ^56^ ^]^ The concept involves the use of upconverting nanoparticles (UCNPs) as transducers of NIR light, which re‐emit UV light, causing rotation of the motor and consequent change in the optical properties of the material (**Figure** **5**a).^[^ ^57^, ^58^, ^59^ ^]^ As UCNPs, we used doped NaYF_4_ nanoparticles^[^ ^60^ ^]^ (see Sections S1 and S2, Supporting information). The emission spectrum of the applied UCNPs showed significant overlap with the motor's absorption spectrum, which is essential for the radiative energy transfer mechanism (Figure S14, Supporting information). Subsequently, we confirmed that NIR light can induce rotation of motor **1** in an acetonitrile solution in the presence of UCNPs (4.3*10^−5^ M motor with 3 mg/L UCNPs, Figure S15, Supporting information) and that the nanoparticles do not affect the chirality of the motor (Figure S16, Supporting information). We then prepared a cholesteric mixture containing 4.5 wt.% of the motor and doped with 2 wt.% UCNPs. The cholesteric layer with a planar alignment prepared between two glass slides reflects right–circularly polarized light with the band centered at 600 nm. Irradiation of the layer with an NIR laser beam (diameter ≈2 mm) leads to a gradual unwinding of the right‐handed supramolecular helix, accompanied by a bathochromic shift of the reflection band until it disappears due to the complete unwinding of the helix (Figure 5b). Further NIR irradiation causes twisting of the left‐handed helical structure until the photostationary state is reached after 25 min of irradiation, characterized by the reflection of blue light with left‐handed circular polarization, which is clearly visible in Figure 5c. It is worth noting that infrared irradiation of the control mixture without UCNPs did not induce any shift of the reflection band (Figure S17, Supporting information), which again supports the mechanism of NIR‐light‐activated inversion.

## 6 Conclusion

Utilizing intrinsically chiral molecular motors has shown unique advantages for applications in dynamic and smart optical devices with fully reversible remote control by light. Light‐driven intrinsically chiral molecular motors are superb candidates for controlling complex behavior in LC materials. Our design shows that a robust and highly photoefficient molecular motor can twist in LCs in a unidirectional manner that allows the generation of four distinct states of the soft material. The optical property of chiral LC layers can be reversibly tuned with light over an exceptionally broad wavelength range. Moreover, due to the high quantum yield of the developed formylated molecular motor core, we presented the first example of NIR‐light‐induced inversion of the supramolecular helical structure of LC phases based on the photo‐isomerization of the molecular motor. Besides a major step toward future applications in photonics, optical devices, and smart materials, this strategy serves as a general design principle providing a highly efficient molecular machine tool for other soft matter responsive systems, such as soft robotics, artificial muscle‐like actuators, and biocompatible materials powered by a NIR‐light‐triggered molecular motion.

## 7 Experimental Section

Synthetic procedures, full characterization of the compounds, and experimental and computational procedures can be found in the Supporting Information.

CCDC 2489663 contains the supplementary crystallographic data for this paper. These data can be obtained free of charge from The Cambridge Crystallographic Data Centre via, https://www.ccdc.cam.ac.uk/data_request/cif.

## Conflict of Interest

The authors declare no conflict of interests.

## Author Contributions

J. S. and A. R. contributed equally to this work. J.S. did conceptualization, investigation, methodology, formal analysis, data curation, Writing – original draft, and Writing – review & editing. A. R. did conceptualization, investigation, methodology, formal analysis, data curation, Writing – original draft; and Writing – review & editing. G.L. did investigation, visualization, and Writing – review & editing. B.L.F. did conceptualization, supervision, funding acquisition, and Writing – review & editing. J.S. and A.R. contributed equally to this work.

## Supporting information



Supporting Information

## Data Availability

The data that support the findings of this study are available in the supplementary material of this article.
