# Supplementary material for: Quadruple Helix Inversions of Liquid Crystals Orchestrated by Direct Chirality Transmission of an Intrinsically Chiral Light‐Driven Molecular Machine
Source: Adv Mater. 2025 Oct 8;38(4):e13156. doi: 10.1002/adma.202513156 (PMC12810663; doi:10.1002/adma.202513156)
Supplement: Supplementary file 1 — Supporting Information [file ADMA-38-e13156-s001.pdf]

ADVANCED MATERIALS

Supporting Information

for *Adv. Mater.*, DOI 10.1002/adma.202513156

Quadruple Helix Inversions of Liquid Crystals Orchestrated by Direct Chirality Transmission of an Intrinsically Chiral Light-Driven Molecular Machine

*Jinyu Sheng, Alexander Ryabchun, Guiying Long and Ben L. Feringa**

Quadruple Helix Inversions of Liquid Crystals Orchestrated by Direct Chirality Transmission of an Intrinsically Chiral Light-Driven Molecular Machine

Jinyu Sheng, [1,2,†] Alexander Ryabchun,[1,†] Guiying Long, [1] Ben L. Feringa[1*]

[1] Stratingh Institute for Chemistry, University of Groningen, Nijenborgh 3, 9747 AG Groningen, the Netherlands
[2] College of Chemistry, Chemical Engineering and Materials Science, Soochow University, Suzhou, Jiangsu 215123, China.
[†]These authors contributed equally
[*]Corresponding author: Prof. B. L. Feringa (b.l.feringa@rug.nl)

Contents

1. General Information 3
Liquid crystal samples preparation. 4
Reagents for UCNP synthesis. 4
Sample preparation (with UCNPs addition). 4
2. Synthesis and Characterizations of UCNPs and motors 5
Synthesis of 25 nm $NaYF_4$:75%Yb^{3+},0.5%Tm^{3+} bare cores 5
Synthesis of 35 nm $NaYF_4$:75%Yb^{3+},0.5%Tm^{3+} @$NaYF_4$ core-shell nanoparticle 5
3. Synthesis and characterization of M1 6
NMR data of M1 7
HRMS of M1 8
4. Photoisomerization of M1 by UV/Vis spectroscopy. 9
5. Kinetic studies of M1 in solution. 10
6. Kinetic studies of M1 in liquid crystals. 11
7. Alignment of molecular motor M1 in liquid crystals 12
8. Quantum yield determination of M1 14
9. Irradiation cycles of M1 in solution by ^{1}H NMR measurements. 17
Overlap of the emission spectrum of UCNP and Absorption spectra of motors 17
UV spectra of (R, P)-Z_S-M1 in solution under exposure of a 980 nm laser with UCNPs. 17
10. Control experiments for UCNP enabled NIR light activation 18
CD spectra of M1 with/without UCNPs 18
UV spectra of M1 in LC under exposure of a 980 nm laser without UCNPs. 18
11. Measurements of helical twisting power 19
12. References 31

1. General Information

All reagents were obtained from commercial sources and used as received without further purification. Dry solvents were obtained from an MBraun solvent purification system. Column chromatography was performed on a Reveleris X2 flash chromatography system. TLC: silica gel 60, Merck, 0.25 mm. High resolution mass spectrometry (ESI or APCI-MS) was performed on a LTQ Orbitrap XL spectrometer with ESI or APCI ionization. NMR spectra were recorded on Varian AMX400 (^{1}H: 400 MHz, ^{13}C: 101 MHz). Chemical shifts are quoted in parts per million (ppm) relative to the residual solvent signal (for $CDCl_3$ δ 7.26 for ^{1}H, δ 77.16 for ^{13}C and for CD_2Cl_2 δ 5.32 for ^{1}H, δ 53.84 for ^{13}C). For ^{1}H NMR spectroscopy, the splitting pattern of peaks is designated as follows: s (singlet), d (doublet), t (triplet), m (multiplet), br (broad), or dd (doublet of doublets). UV/Vis absorption spectra were measured on a Hewlett-Packard 8454 diode array spectrometer in a 1 cm quartz cuvette. All the irradiation experiments in solutions were performed using Thorlab LEDs (M365FP1, M455F3). Solvents used for spectroscopic studies were of spectroscopic grade (UVASOL, Merck) or from a solvent purification system. Circular dichroism (CD) spectra in solution were recorded with a JASCO J-815 spectropolarimeter at room temperature. CD spectra of the cholesteric layers were measured using a HP8454 UV-Vis spectrophotometer equipped with achromatic linear polarized and λ/4-plate positioned at ±45° with respect to polarization plane. Polarized light absorbance spectra were recorded by AVASPEC-ULS2048CL-EVO (Avantes) spectrometer equipped with Glan-Taylor polarizer on a rotational mount (ELL14K, Thorlabs). Irradiation of liquid crystal samples were performed using mounted LEDs (Thorlabs) at 365 nm and 455 nm. Light intensities were measured with light powermeter PM100A (Thorlabs).

Helical twisting powers (HTPs) of molecular motor in different liquid crystal hosts were determined by the Grandjean-Cano wedge method.[1] The handedness of cholesteric helical structure was determined using a rotatable analyzer as previously reported.[2] Optical imaging and measurements were performed using polarized optical microscope (POM) Eclipse LV100N-POL (Nikon, Japan) and Nis-Element-D software.

Liquid crystal samples preparation.

The doped liquid crystal mixtures were prepared by dissolving the necessary amounts of molecular motor and nematic liquid crystal (MLC6816, 5CB, E7, ZLI1083 all from Merck) in dichloromethane, followed by solvent evaporation and drying in vacuum. For helical twisting power measurements, we prepared the mixtures containing 1 wt% of molecular motor while 4.3 wt% mixtures were used for measuring reflection colors. These mixtures were introduced into wedge or planar cells by capillary forces at room temperature. The wedge cells ($tan\theta = 0.0115$) were purchased from E.H.C. Co. Ltd. (Japan). Home-made quartz cells promoting planar unidirectional alignment were used for spectroscopic investigations. The rubbed poly(vinylalcohol) layers coated on both quartz substrate were applied to promote unidirectional liquid crystal alignment. Thickness of the cells was 6 μm and was controlled by Mylar spacers.

Reagents for UCNP synthesis.

$LnCl_3$•$6H_2O$ (Ln: Er, Yb, Y, 99.9% trace metals basis), Ln_2O_3 (Ln: Y, Tm, Yb, Nd, 99.9% trace metals basis), oleic acid (OA), 1-octadecene (ODE, 90%, technical grade), sodium hydroxide (NaOH, 97%), trifluoroacetic acid (TFA, 99%), sodium trifluoroacetate (Na-TFA, 98%), *N*, *N*-dimethylformamide (DMF 99%), and Nitrosonium tetrafluoroborate ($NOBF_4$ 95%) were obtained from Sigma Aldrich. Acetone (>99%), ethanol (96%), and cyclohexane were purchased from VWR chemicals. Ammonium fluoride (NH_4F, 98%+ extra pure) was purchased from Acros organics.

Sample preparation (with UCNPs addition).

Stock solution of **M1** (in MeCN), and **UCNP**s (15 mg/mL, in MeCN) were prepared. From them, a solution of **M1/UCNPs** with different concentrations was prepared in MeCN (2mL) in a quartz cuvette without degassing procedure needed. Near infrared continuous wave laser (MDL-H-980nm, ordered from Changchun New Industries Optoelectronics Tech. Co.) were used as light sources for motor rotation and positioned at a fixed distance to the cuvette (Figure S1A), the reaction was monitored by UV-Vis absorption spectra. To irradiate liquid crystal layers laser light coupled to 1mm optical fiber and collimated to a beam of 2 mm in diameter (Figure S1B). Total light power was 3.2W (or approx. 1 W mm^{-2}).

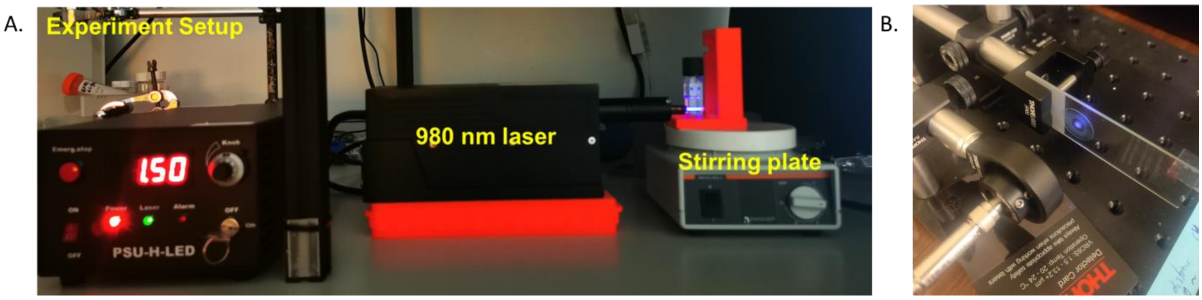

Supplementary Fig. S1. Experimental set-up for the NIR light-induced unidirectional motor rotation in solution (A) and in liquid crystal (B).

2. Synthesis and Characterizations of UCNPs and motors

Synthesis of 25 nm $NaYF_4$:75%Yb^{3+},0.5%Tm^{3+} bare cores

The cores $NaYF_4$:75%Yb^{3+}, 0.5%Tm^{3+} were synthesized by a well-developed thermal decomposition method.[3] Firstly, 0.75 mmol $YbCl_3·6H_2O$, 0.005 mmol $TmCl_3·6H_2O$, and 0.245 mmol $YCl_3·6H_2O$ were added to a mixture of 6 mL oleic acid (OA, 90%) and 15mL 1-octadecene (ODE, 90%) in a 100mL three-necked round-bottom flask. Then, the chlorides were dissolved at 160°C for 30 min under nitrogen flow. After the solution was cooled down to room temperature, 2.5 mmol NaOAc and 4 mmol NH_4F were added. The mixture was heated to 100°C for 1 h in vacuum until the powders dissolved. Subsequently, the temperature of the resulting solution was quickly increased to 300 °C and maintained at that temperature for 90 min in a nitrogen atmosphere. The mixture was cooled down to room temperature, and the products were collected by centrifugation at 8000 rpm for 10 min with ethanol twice, and finally dispersed in 4 mL of cyclohexane.

Synthesis of 35 nm $NaYF_4$:75%Yb^{3+}, 0.5%Tm^{3+} @$NaYF_4$ core-shell nanoparticle

Core-shell nanoparticle synthesis following a well-developed method.[4] Typically, 1 mmol $Y(CF_3COO)_3·3H_2O$ and 1 mmol CF_3COONa were mixed with 3 mL oleic acid and 7.5 mL 1-octadecene in a 100 mL flask. The solution was heated to 150 °C to form a homogenous shell precursor solution and then cooled down to room temperature. After that, 1 mL of the prepared bare core particles in cyclohexane ($NaYF_4$:75%Yb^{3+},0.5%Tm^{3+}, 0.25 mmol) was added into a 100 mL three-neck round-bottom flask followed by 3 mL OA and 7.5 mL ODE addition. The solution was then heated up to 100 °C in vacuum for 30 min to remove cyclohexane and residual air. Next, the solution was heated up to 300 °C under a nitrogen atmosphere and stirred for 5 min. The as-obtained $NaYF_4$ shell precursors (10.5 mL) were injected into the solution during four 15-min intervals. After the injection of all shell precursors, the mixture was allowed to ripen for 45 min before cooling down. The core-shell UCNPs ($NaYF_4$:75%Yb^{3+}, 0.5%Tm^{3+}@$NaYF_4$) were collected by centrifugation with ethanol twice and redispersed in 5 mL cyclohexane.

To facilitate the transfer of UCNPs from a hydrophobic medium (e.g., cyclohexane) to a hydrophilic medium (e.g., MeCN), a ligand removal strategy is often employed due to its versatility. Here, 2 mL of the as-synthesized UCNP and 2 mL of DMF are combined in a 10 mL vial, followed by the addition of 10 mg of Nitrosonium tetrafluoroborate ($NOBF_4$). The resulting mixture was sonicated for 20 mins and then centrifuged at 10000 rpm for 10 min to precipitate the quasi-ligand- free UCNPs in DMF. The UCNPs can then be easily redispersed in 2 mL of MeCN.

3. Synthesis and characterization of M1

Both enantiopure and racemic precursors, i.e., ***S***1 (racemic or ee:98%) and ***S***2 were obtained according to our previous developed methods.[5] Enantiopure ***S***2 was obtained by using enantiomeric S1 as the starting material.

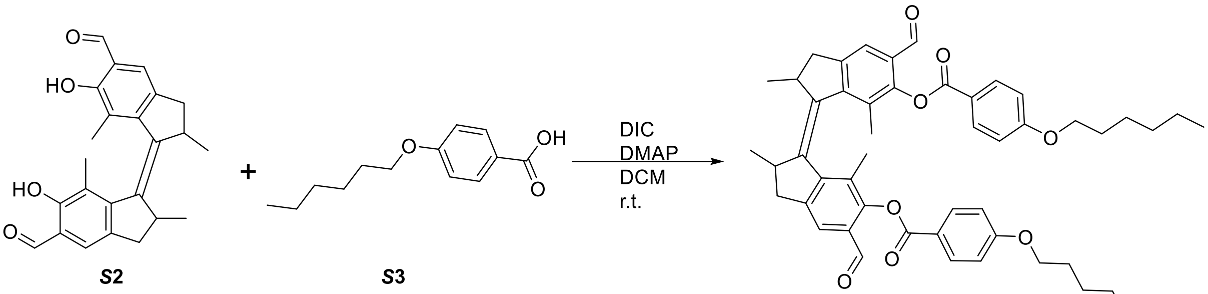

(Z)-5,5'-diformyl-2,2',7,7'-tetramethyl-2,2',3,3'-tetrahydro-[1,1'-biindenylidene]-6,6'-diyl bis(4-(hexyloxy)benzoate)

Under a N_2 atmosphere, (*P*,*P*)-***S*2** (20.0 mg, 0.05 mmol, 1.0 equiv.), **S3** (28.0 mg, 0.13 mmol, 2.5 equiv.) and DMAP (8.0 mg, 0.07 mmol, 1.4 equiv.) was dissolved in dry CH_2Cl_2 (3.0 mL). DIC (100 mg, 0.8 mmol, 16.0 equiv.) was added to the solution, and the mixture was stirred at room temperature for 6 h. Water (5 mL) was added and the mixture extracted with EtOAc (3*10 mL). The combined organic layers were washed with brine (20 mL), dried over Na_2SO_4 and concentrated under vacuum. The crude yellow solid was purified by column chromatography (SiO_2, pentane:EtOAc = 10:1 to 5:1) to afford pure *Z*-**M1** as a yellow solid (35.3 mg, 0.045 mmol, 90%).

Note: The reaction mixture should be covered with Aluminium foil and all work-up steps should avoid long-time room light illumination to avoid isomerization of the product.

Racemic ***S***2 was used for the synthesis of racemic *Z*-**M1**s applying the same protocol.

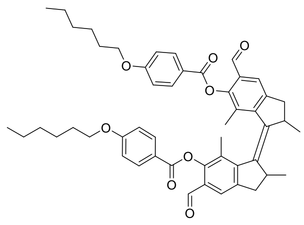

^{1}H NMR (400 MHz $CDCl_3$) δ 10.08 (s, 2H), 8.17 – 8.12 (m, 4H), 7.67 (s, 2H), 7.03 – 6.98 (d, 4H), 4.06 (t, *J* = 6.5 Hz, 4H), 3.51 – 3.44 (m, 2H), 3.30 (dd, *J* = 15.1, 6.3 Hz, 2H), 2.61 (d, *J* = 15.1 Hz, 2H), 1.86 – 1.79 (m, 4H), 1.53 (s, 6H), 1.48 (d, *J* = 7.8 Hz, 4H), 1.37 (dt, *J* = 7.4, 3.8 Hz, 8H), 1.15 (d, *J* = 6.8 Hz, 6H), 0.94 – 0.90 (t, 6H).

^{13}C NMR (101 MHz, $CDCl_3$) δ 189.0, 164.6, 164.0, 151.6, 148.2, 143.7, 143.5, 132.6, 130.3, 127.7, 122.1, 120.8, 114.6, 68.5, 42.8, 39.9, 31.7, 29.9, 29.2, 25.8, 22.7, 20.1, 14.7, 14.2.

HRMS (ESI pos) calcd $C_{50}H_{56}O_8Na$ $[M+Na]^+$: 807.3867, found 807.3846.

NMR data of M1

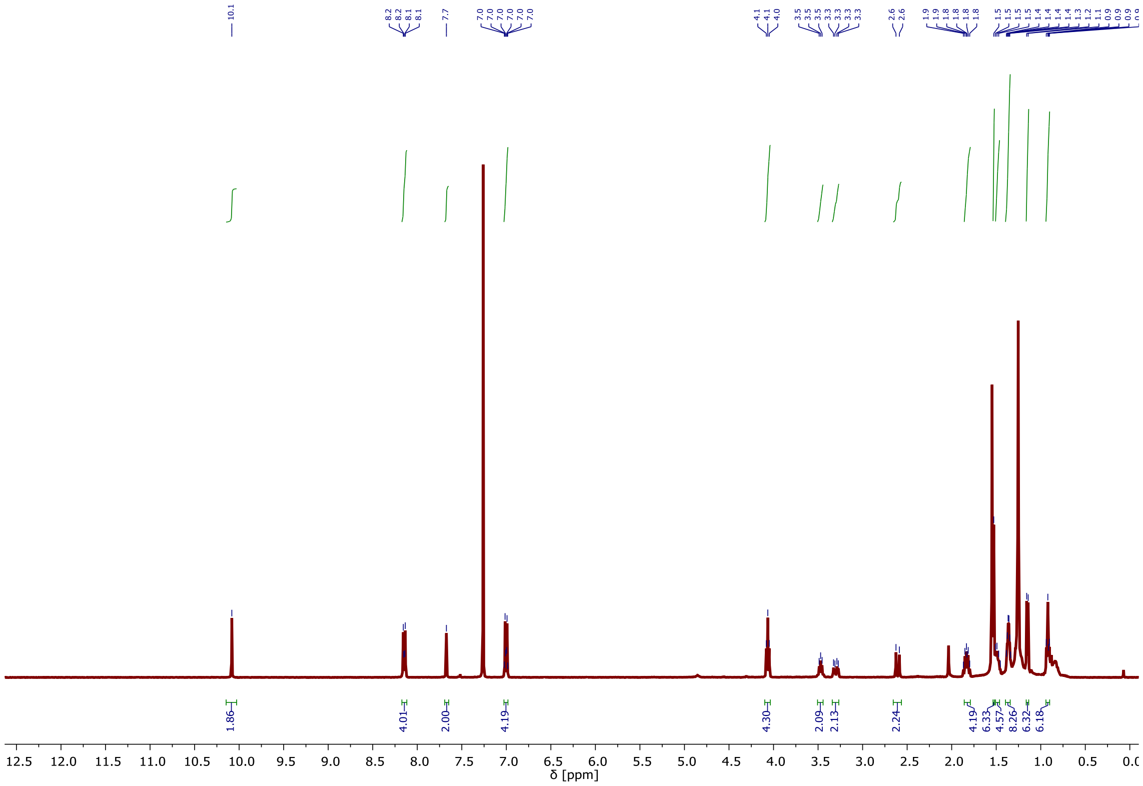

Supplementary Fig. S2. ^{1}H NMR (400 MHz, $CDCl_3$, 20 ºC) spectrum of *Z*-M**1**$_S$.

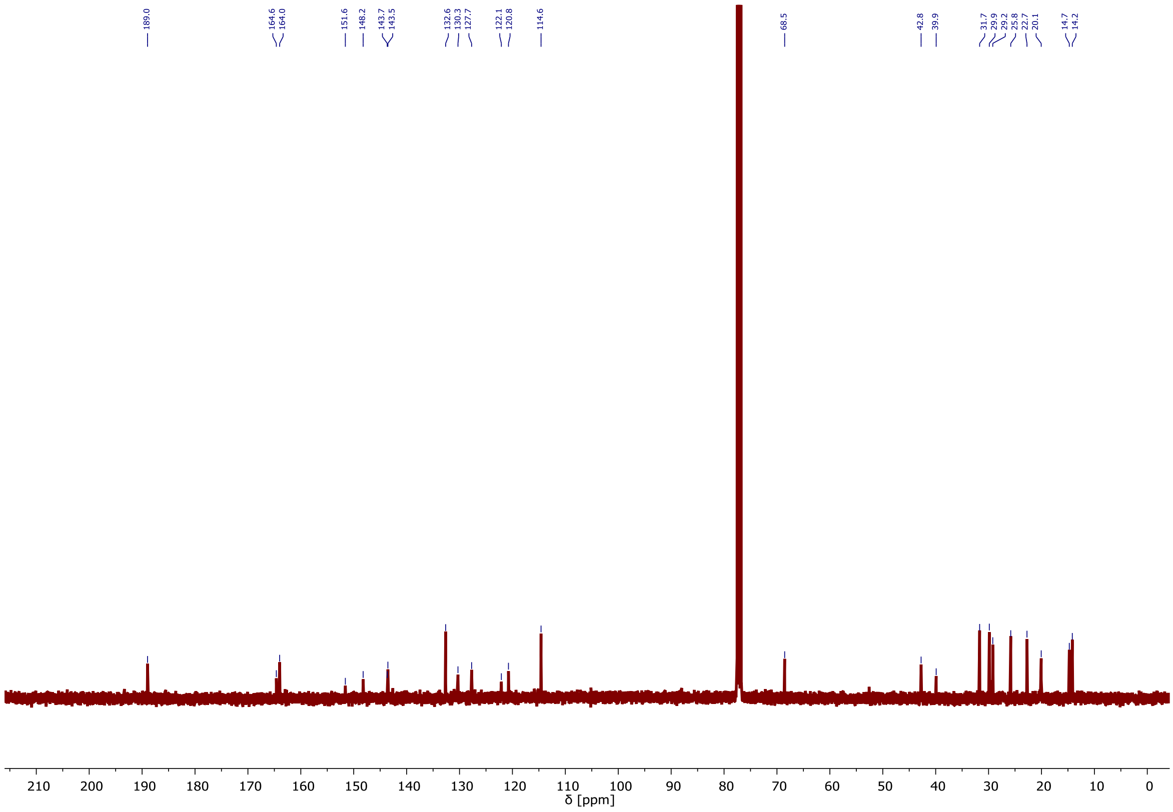

Supplementary Fig. S3. ^{13}C NMR (101 MHz, $CDCl_3$, 20 ºC) spectrum of *Z*-M**1**$_S$.

HRMS of M1

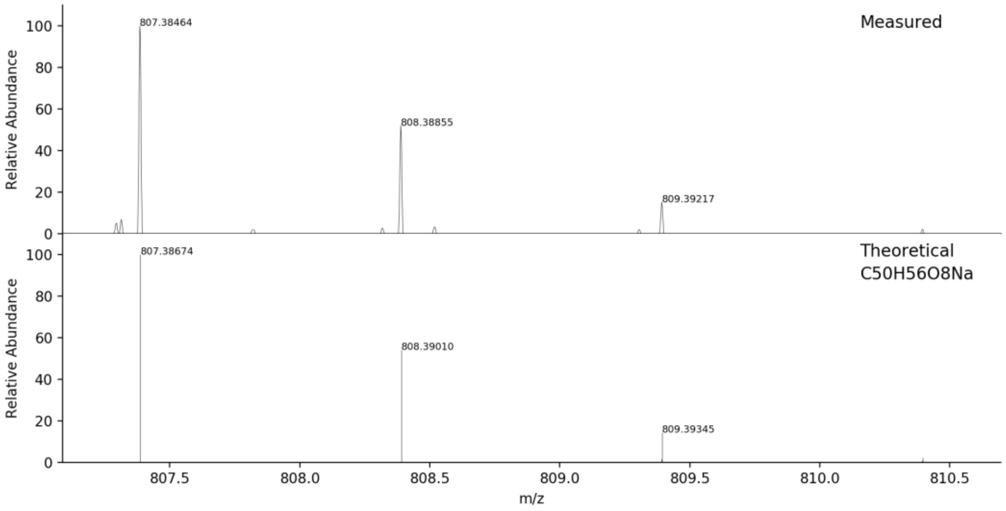

Supplementary Fig. S4. HRMS data of *Z*-M**1**$_S$.

4. Photoisomerization of M1 by UV/Vis spectroscopy.

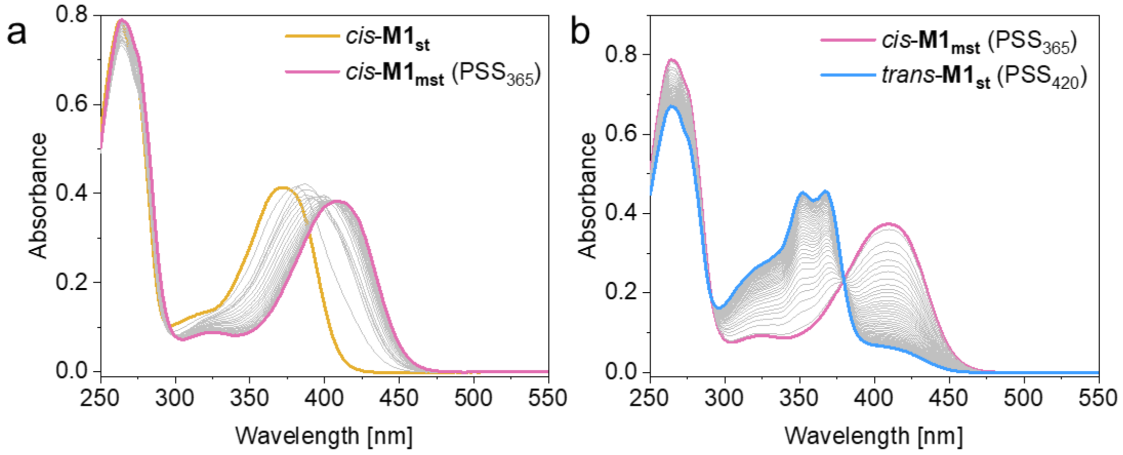

Supplementary Fig. S5. a. UV/vis electronic absorption spectra of motor *Z*-M**1**$_S$ (yellow line) upon irradiation at 365 nm to *Z*-M**1**$_M$ (pink line) to PSS (DCM, r.t., 1.0*10^{-5} M). **b.** UV/vis electronic absorption spectra of *Z*-**M1**$_M$ (pink line) upon irradiation at 420 nm to *E*-**M1**$_S$ (blue line) to PSS.

5. Kinetic studies of M1 in solution.

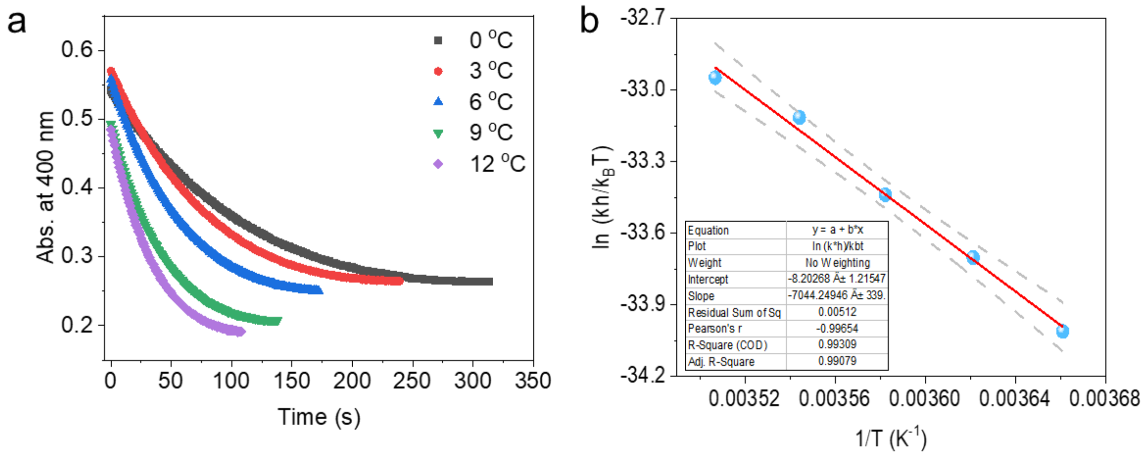

Supplementary Fig. S6. a. Kinetic decay monitored at 400 nm from *E*-**M1**$_{\mathbf{M}}$ to *E*-**M1**$_{\mathbf{S}}$ in CH_2Cl_2. **b.** Eyring plot of THI step from motor *E*-**M1**$_{\mathbf{M}}$ to *E*-**M1**$_{\mathbf{S}}$ in CH_2Cl_2. Dashed lines indicate 95% confidence intervals.

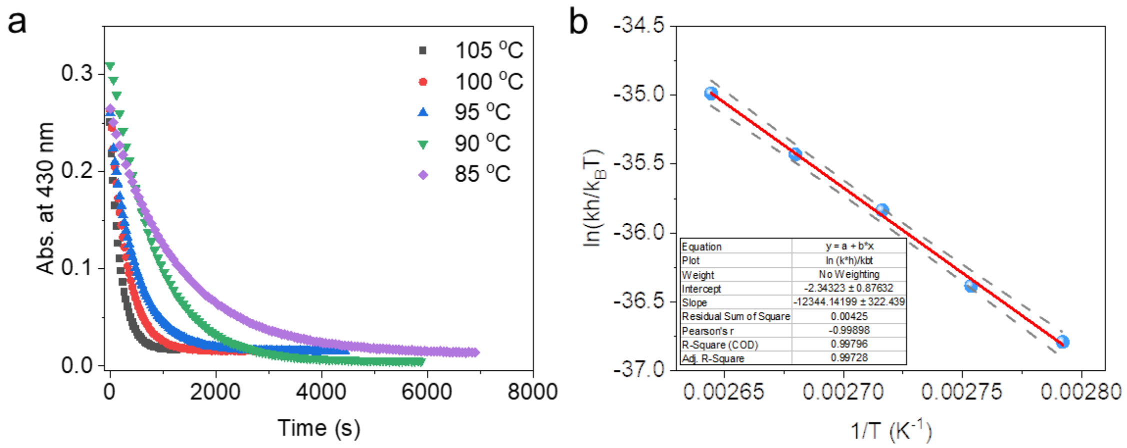

Supplementary Fig. S7. a. Kinetic decay monitored at 430 nm from *Z*-**M1**$_{\mathbf{M}}$ to *Z*-**M1**$_{\mathbf{S}}$ in DMSO. **b.** Eyring plot of THI step from motor *Z*-**M1**$_{\mathbf{M}}$ to *Z*-**M1**$_{\mathbf{S}}$ in DMSO. Dashed lines indicate 95% confidence intervals.

6. Kinetic studies of M1 in LC.

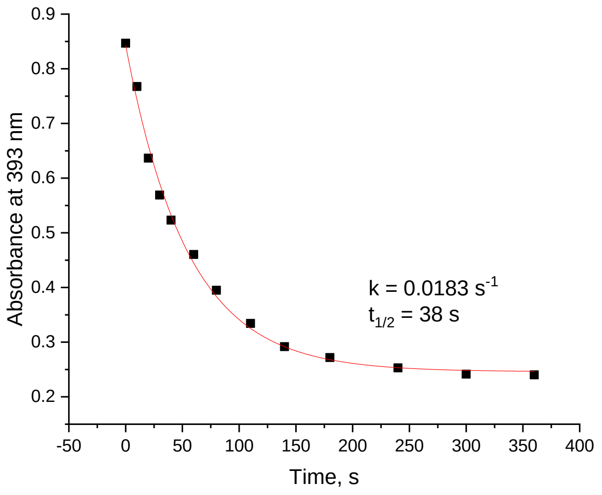

Supplementary Fig. S8. Kinetic decay monitored at 393 nm from ***E*-M1$_M$** to ***E*-M1$_S$** in LC (4.3wt% in ZLI1083). The ***Z*-M1$_S$** was irradiated at 365 nm for 2 s followed by kinetic measurements at 20 °C.

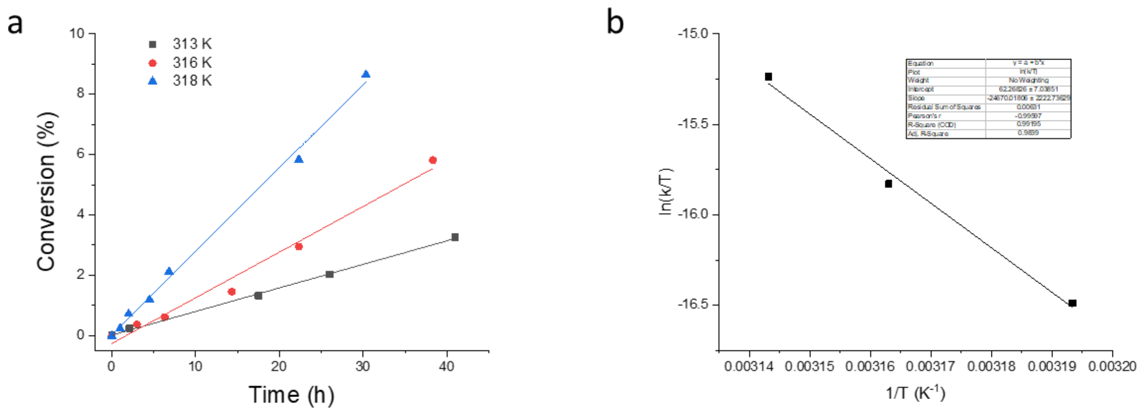

Supplementary Fig. S9. a. Kinetic curves of ***Z*-M1$_M$** to ***Z*-M1$_S$** THI process in ZLI1083 LC host. Conversion has been calculated based on shift of selective light reflection wavelength of planarly aligned layer of ZLI1083 doped with 4.3wt% ***Z*-M1$_S$**. ***Z*-M1$_M$** was generated by 365 nm UV exposure. **b.** Eyring plot of the THI step in LCs.

7. Alignment of molecular motor M1 in liquid crystals

Calculations

The ground state structures of motor were optimized for each of the forms involved in the rotary cycle, by using density functional theory with ωB97X-D4[6] hybrid functional and def2-TZVPP basis set. We performed a vertical excitation calculation for each of the optimized geometries, by using the time-dependent density functional theory method (TD-DFT) without Tamm-Dancoff approximation, with the same functional and basis set using the conductor-like polarizable continuum solvent model CPCM(acetonitrile)[7] considering 30 singlet transitions. Orientations of $S_0 \rightarrow S_1$ transition dipole moments (TDMs) (Fig. S10A) as well as molecular axis (minimum moment of inertia axis) were extracted from those calculations to interpret the spectral data on motor alignment in the liquid crystal network (Fig. S10B, C). All calculations have been performed using the Orca 5.0.4 package.[8]

Measurements

We investigated the alignment of molecular motor in three states in liquid crystal (ZLI183) by means of linearly polarized light UV-Vis spectroscopy. This technique allows estimating the orientation of the electronic transition dipole moment (TDM) in the motor (calculated by TD-DFT, see above) and, consequently, of the molecular motor itself with respect to the orientation of liquid crystal molecules. The 10 μm quartz cell promoting unidirectional molecular alignment (rubbed polyimide layers) was prepared. 1wt% mixture of racemic *Z*-**M1**$_S$ motor in ZLI1083 was used. For measuring a polarized light absorbance, the spectrometer was equipped with a polarizer (Glen-Taylor prism) mounted on a rotatable stage to change the angle of polarization of the light in 10-degrees increments.

As shown in **Supplementary Fig. S10**, the polarized absorbance of the molecular motors in the E_s state was significantly larger parallel to the LC director than in the perpendicular direction, indicating that the TDM (long molecular axis, minimum moment of inertia (MOI)) of molecular motor was mainly aligned along the LC alignment axis as evidenced by the polar plot (Figure S10C). TDMs of both Z_M and Z_S were found to be mainly aligned perpendicular to the LC orientation. TDM in these states of the motor is rotated approx. 90 degrees in respect to rigid motor's halves indicating MOI orientation along the LC alignment. Interestingly, the quality of molecular alignment of E_S and Z_S states is identical and (but in opposite directions) as indicated by spectroscopic order parameter (S) (Fig. S10C). However, the Z_M state is better aligned as evidenced by high S value.

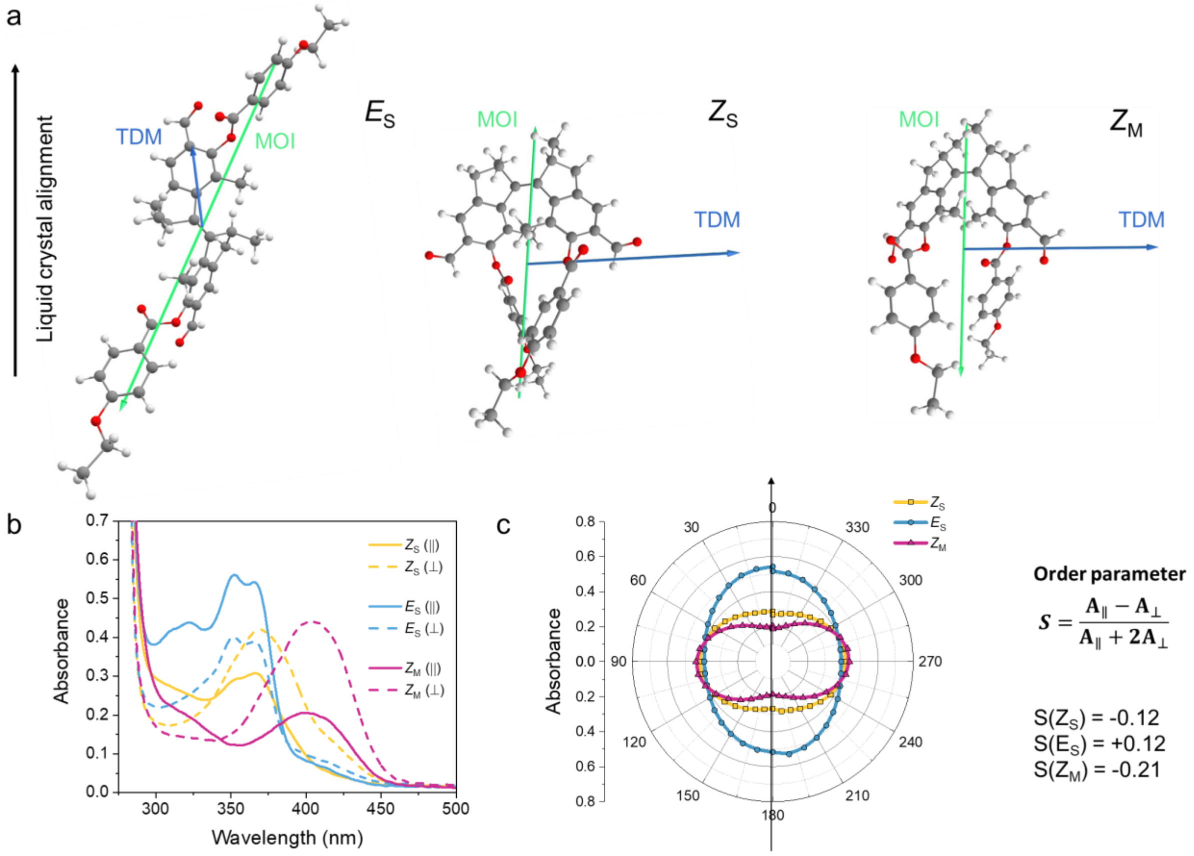

Supplementary Fig. S10. a. Scheme demonstrating an alignment of molecular motor **M1** (DFT optimized geometry) with respect to the liquid crystals alignment. TDM – transition dipole moment, MOI - minimum moment of inertia. **b.** Polarized absorbance spectra of molecular motor in unidirectionally aligned LC medium. **c.** Angular dependence as well as order parameter (S) of **M1** in different states. Equation to estimate S is provided in panel C, where $A_{||}$ and $A_{\perp}$ are the absorbance of light polarized parallel and perpendicular to the direction of the liquid crystal alignment. S = 0 indicates the absence of any order, and S = 1 (S = -0.5) indicates perfect order along (perpendicular) to LC alignment. The direction of LCs alignment is indicated with black arrow. Thickness of the quartz cell was 10 mm, concentration of M1 in ZLI1083 was 1 wt%.

8. Quantum yield determination of M1

Ferrioxalate Chemical Actinometry

A modification of a standard protocol was applied for the determination of the photon flux.[9,10] An aqueous H_2SO_4 solution (0.05 M) containing freshly recrystallized $K_3[Fe(C_2O_4)_3]$ (41 mM, 2.0 mL, 1 cm quartz cuvette) was irradiated at 20 °C for a given period of time with exclusion of ambient light with a 365 or a 445 nm LED, under stirring. The solution was then diluted with 1.0 mL of an aqueous H_2SO_4 solution (0.5 M) containing phenanthroline (1 g/L) and NaOAc (122.5 g/L) and left to react for 10 min. The absorption at λ= 510 nm was measured and compared to an identically prepared non-irradiated sample. The experiment was repeated with fresh samples with increasing irradiation times. The concentration of the $[Fe(phenanthroline)_3]^{2+}$ complex was calculated using its molar absorptivity (ε = 11100 M^{-1} cm^{-1}) and considering the dilution. The quantity of Fe^{2+} ions expressed in mol was plotted versus time (expressed in seconds, s) and the slope, obtained by linear fitting the data points to the equation y = ax + b, equals the rate of formation of the Fe^{2+} ion at the given wavelength. This rate can be converted into the photon flux (I) by dividing it by the quantum yield of the $[Fe(C_2O_4)_3]^{2+}$ complex at the wavelength of interest (Φ^{365nm} = 1.21)[5] and by the probability of photon absorption of the Fe^{3+} complex (approximated to **1** for 365 nm LED as we were working in total absorption regime). The obtained molar photon fluxes were $I^{365nm} = 3.95 \cdot 10^{-5}$ mmol s^{-1}.

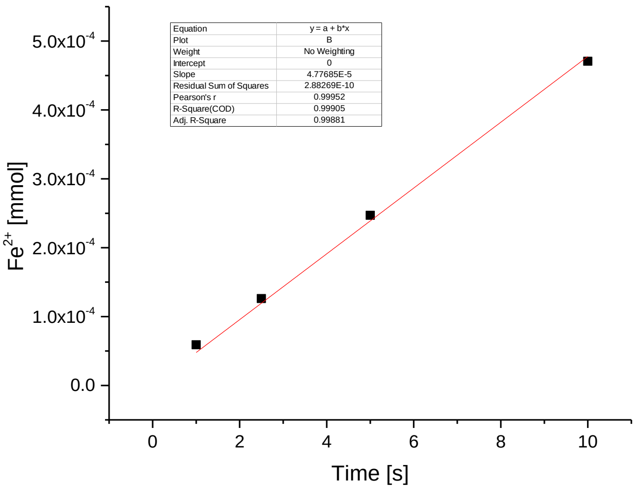

Supplementary Fig. S11. Linear fitting of the Fe^{2+} moles generated upon irradiation of the $[Fe(C_2O_4)_3]^{2+}$ complex 365 nm at different irradiation times.

Quantum yields determination by UV/Vis method in solution

Solutions of the stable state of a specific motor (S) in acetonitrile were irradiated with an LED of a selected wavelength (*i.e.* 365 nm) to lead to the formation of the metastable state (M). The spectra were collected following the evolution of the absorption at the wavelength of irradiation; the average absorbance value recorded between 600-800 nm was subtracted to the datapoints. The data was subsequently fitted using COPASI 4.29[11] following the same approach developed by Stranius & Börjesson.[6] Equiv. 14 in the original article

$$\frac{d[S]}{dt} = -\frac{QY_{SM} \cdot I \cdot \beta_S(t)}{N_A \cdot V} + \frac{QY_{MS} \cdot I \cdot \beta_M(t)}{N_A \cdot V}$$

was used to determine both approximated QYs (QY_{SM} for the formation of the metastable state form the stable and QY_{MS} for the opposite photochemical reaction). *I* is the photon flux, previously determined with ferrioxalate actinometry, N_A the Avogadro number, *V* the total volume of the irradiated solution (2 mL) and β the fractions of photons absorbed by either the stable or the metastable state. $\varepsilon_{E\text{-}\mathbf{1S}}$ = 23450 M^{-1} cm^{-1}. The decay was fitted by the ordinary differential equation (ODE) solver present in COPASI, using a Levenberg-Marquardt algorithm with randomized initial conditions. To obtain physically sound results, the boundaries for the QYs values were fixed between $1{\cdot}10^{-6}$ and 1. Using this method, the quantum yield measurement of each switch was performed three times and the reported quantum yields are an average of those measurements. The standard errors on the obtained averages are typically 2-3%.

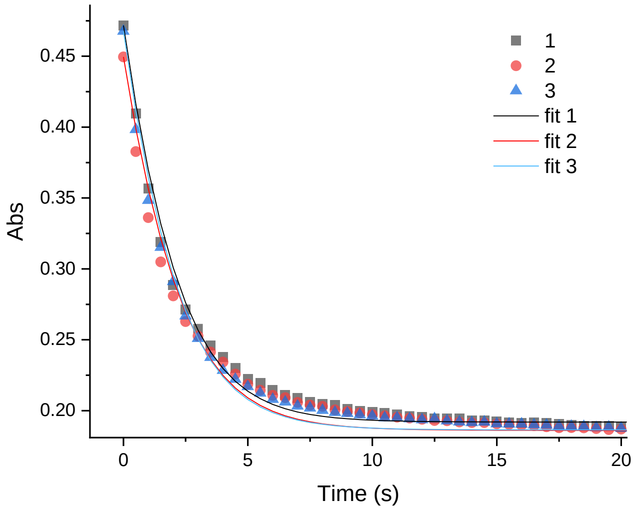

Supplementary Fig. S12. Evolution of the absorption at 365 nm during the irradiation of *E*-**M1**$_S$ in acetonitrile at 365 nm. The solid lines represent the fits obtained with the ODE solver from COPASI.

Quantum yield determination by UV/Vis method in LC phase

The Equiv. 17 in the original article[9] was used to determine approximated QYs in LC cell. *E*-**M1**$_S$ in ZLI1083 liquid crystal cell, irradiation with 365 nm, kinetic traces at 365 nm. Photon flux I^{365nm} =1.5 e^{-5} mmol/s, Area=0.38 cm^2, Qz cell d=10 μm, $\varepsilon_{E\text{-}1S}$ = 47244 M^{-1} cm^{-1}. Note: $\varepsilon_{E\text{-}1S}$ in acetonitrile and LC is different and has to be taken into account. The decay was fitted by the ODE solver present in COPASI, using a Levenberg-Marquardt algorithm with randomized initial conditions.

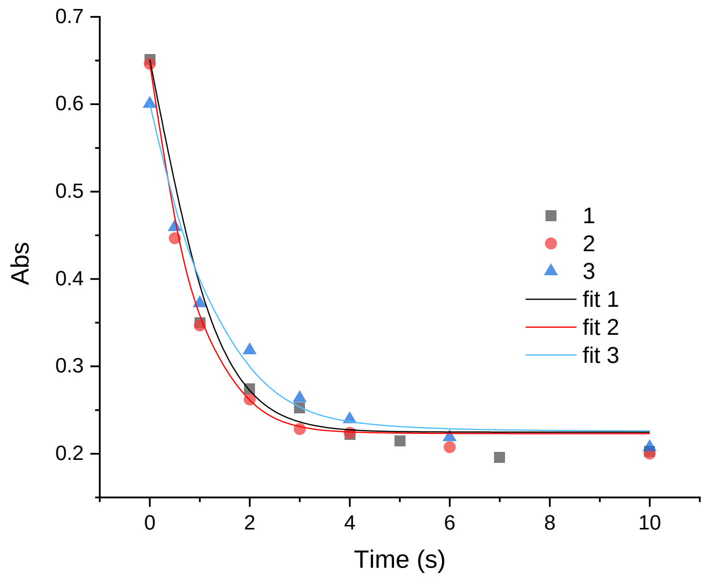

Supplementary Fig. S13. Evolution of the absorption at 365 nm during the irradiation of *E*-**M1**$_S$ in ZLI1083 at 365 nm. The solid lines represent the fits obtained with the ODE solver from COPASI.

9. Irradiation cycles of M1 in solution by ^{1}H NMR measurements.

Overlap of the emission spectrum of UCNP and absorption spectra of motors

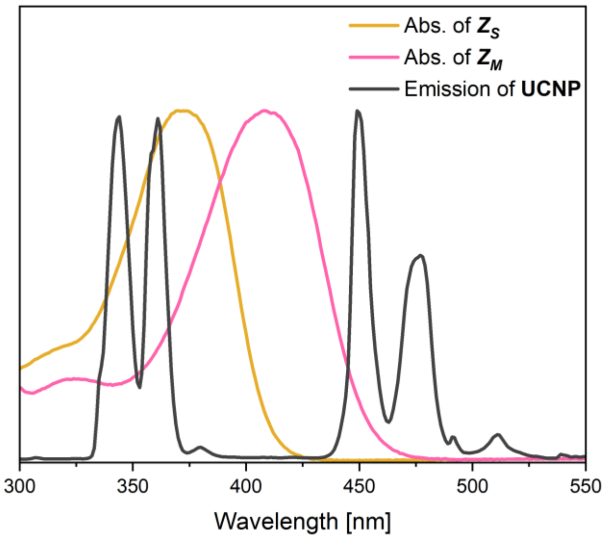

Supplementary Fig. S14. Overlap of the emission spectrum of **UCNP** and absorption spectra of motor **M1**. The normalized *(R, P)-**Z_M*** is generated from the PSS_{365} of an irradiated *(R, P)-**Z_S*** sample.

UV spectra of *(R, P)-Z_S*-M1 in solution under exposure of 980 nm laser with UCNPs.

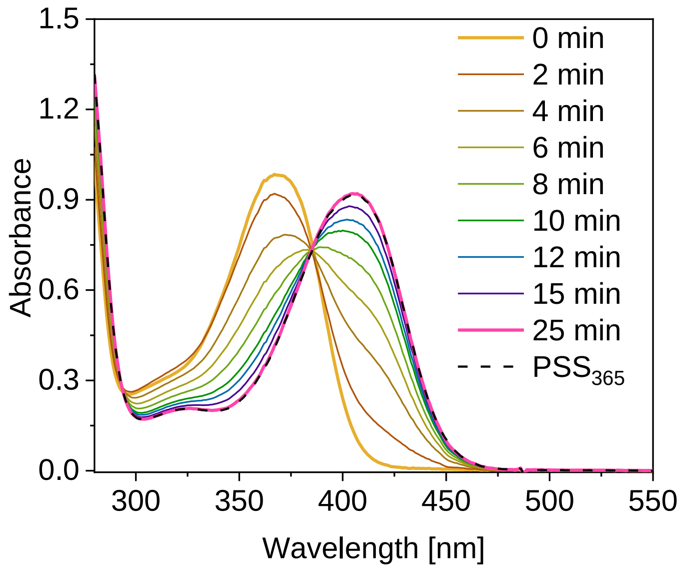

Supplementary Fig. S15. UV spectra of *(R, P)-**Z_S*** (r.t., 4.3*10^{-5} M) in acetonitrile with (3mg/mL UCNPs, bottom) upon irradiation with 980 nm laser to generate *(R, P)-**Z_M*** species. The result shows photoisomerization of *(R, P)-**Z_S*** upon irradiation at 980 nm with UCNPs could reach as high PSS as direct irradiation at 365 nm.

10. Control experiments for UCNP enabled NIR light activation

CD spectra of M1 with/without UCNPs

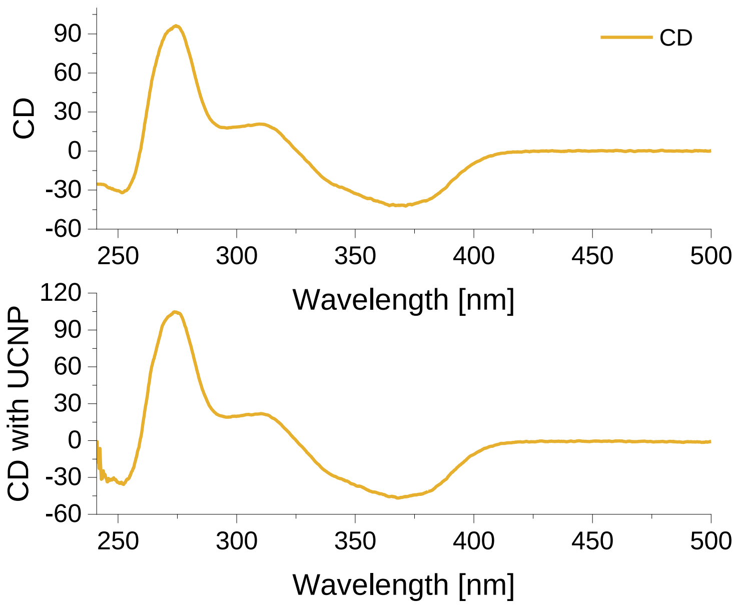

Supplementary Fig. S16. CD spectra of *Z*-**M1**$_S$ (r.t., 4.3*10^{-5} M) in acetonitrile with (3mg/mL UCNPs, bottom) or without UCNPs (top). The result shows no influence of CD signals of *Z*-**M1**$_S$ after UCNPs addition.

UV spectra of M1 in LC under exposure of 980 nm laser without UCNPs.

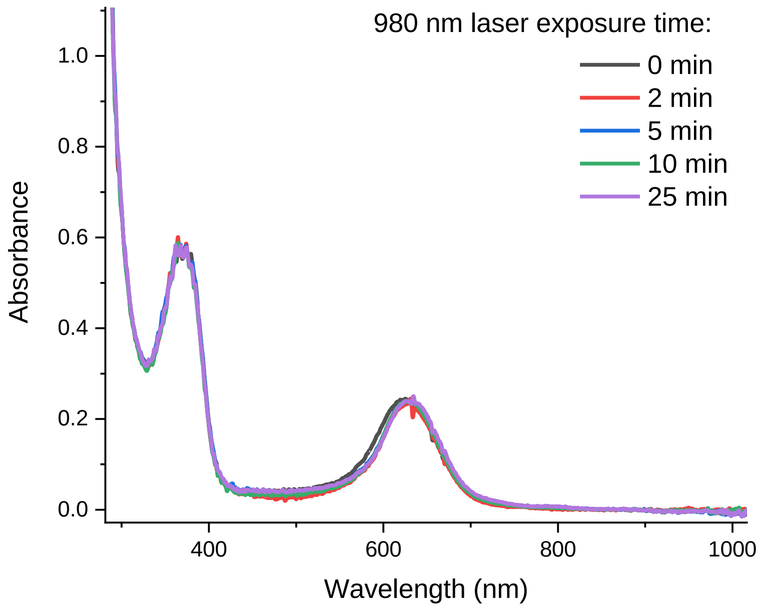

Supplementary Fig. S17. UV spectra of *Z*-**M1**$_S$ (4.5 wt%) in ZLI1083 without UCNPs upon irradiation with a NIR laser.

11. Measurements of helical twisting power

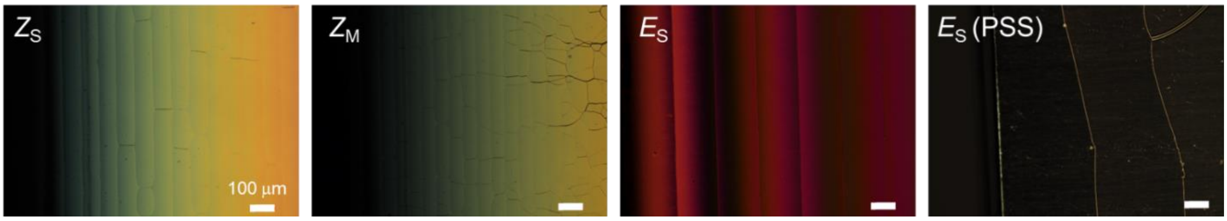

Supplementary Fig. S18. Polarized optical images of wedge cells (*tan*θ = 0.0115) filled with different states of motor **M1** (1 wt.%) dissolved in 5CB liquid crystal. Image of E_S was taken with a red filter to avoid any possible photoreactions. E_S (PSS) corresponds to the Z_M irradiated with 455 nm until photostationary state.

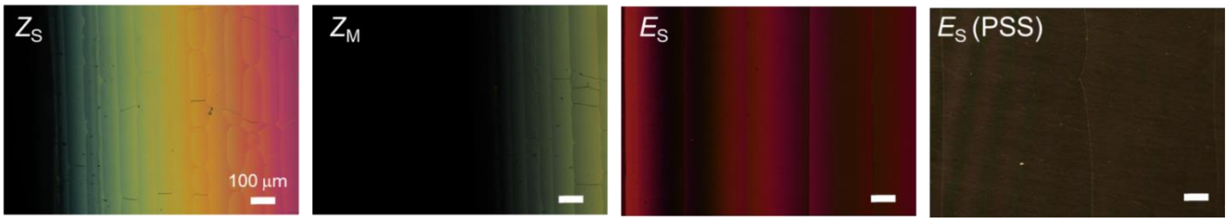

Supplementary Fig. S19. Polarized optical images of wedge cells (*tan*θ = 0.0115) filled with different states of motor **M1** (1 wt.%) dissolved in E7 liquid crystal. Image of E_S was taken with a red filter to avoid any possible photoreactions. E_S (PSS) corresponds to the Z_M irradiated with 455 nm until photostationary state.

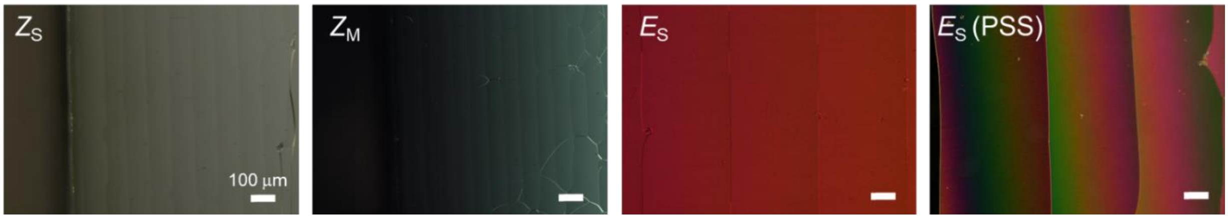

Supplementary Fig. S20. Polarized optical images of wedge cells (*tan*θ = 0.0115) filled with different states of motor **M1** (1 wt.%) dissolved in MLC6816 liquid crystal. Image of E_S was taken with a red filter to avoid any possible photoreactions. E_S (PSS) corresponds to the Z_M irradiated with 455 nm until photostationary state.

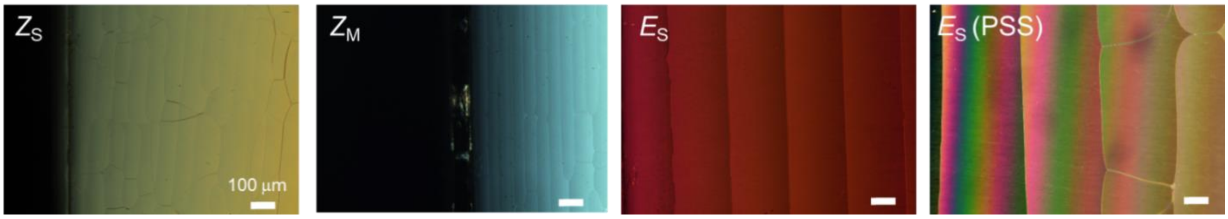

Supplementary Fig. S21. Polarized optical images of wedge cells (*tan*θ = 0.0115) filled with different states of motor **M1** (1 wt.%) dissolved in ZLI1083 liquid crystal. Image of E_S was taken with a red filter to avoid any possible photoreactions. E_S (PSS) corresponds to the Z_M irradiated with 455 nm until photostationary state.

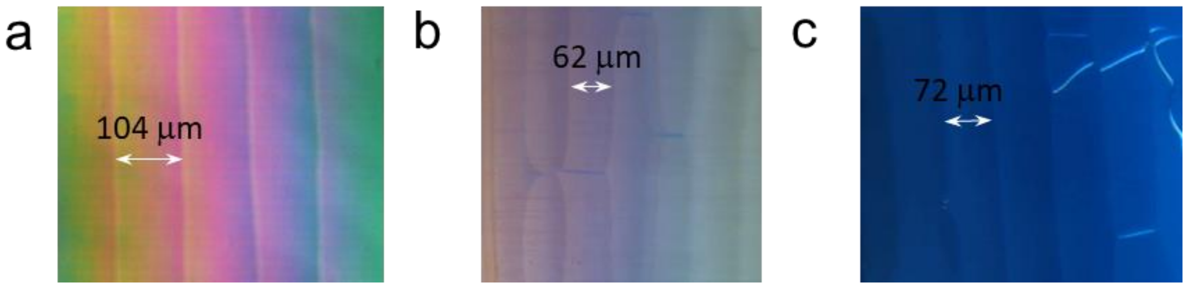

Supplementary Fig. S22. Polarized optical images of wedge cells ($tan\theta = 0.0192$) filled with E_M (1 wt.%) dissolved in E7 (a), ZLI1083 (b), MLC 6816 (v). The E_M state of the motor was prepared by irradiating samples containing Z_S by 365 nm UV light (200 mW/cm^2) for 5 s at 5ºC.

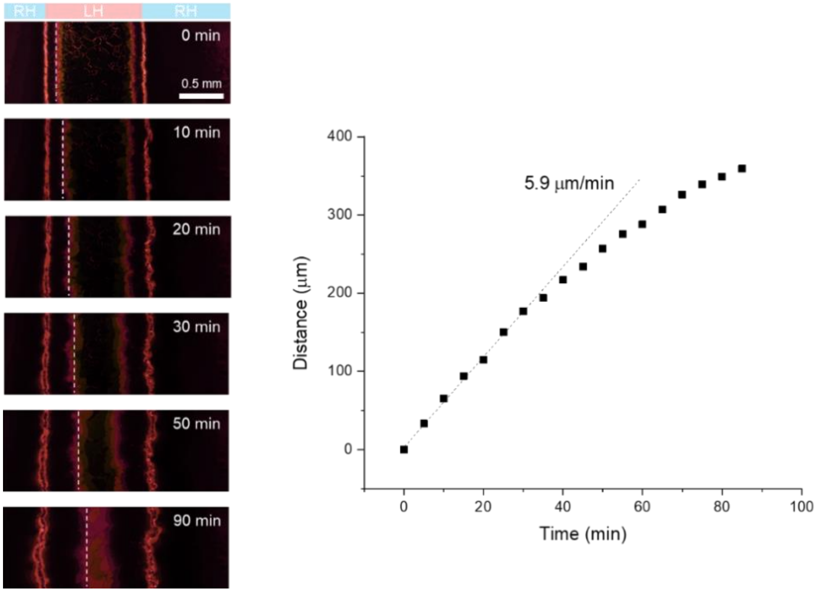

Supplementary Fig. S23. Evaluation of the diffusion controlling stability of the colour patterns. The layer (Z_S 4.3 wt.% in ZLI1083) was irradiated trough a slit (width 970 µm) with UV light forming the area with inverted handedness (left-handed in given case). Over time the set of disclination (defect) lines move (defuse) from the edges to the centre of the exposed area. Plotting lines shift versus time allows to estimate the speed with which colours of the patterns fade away.

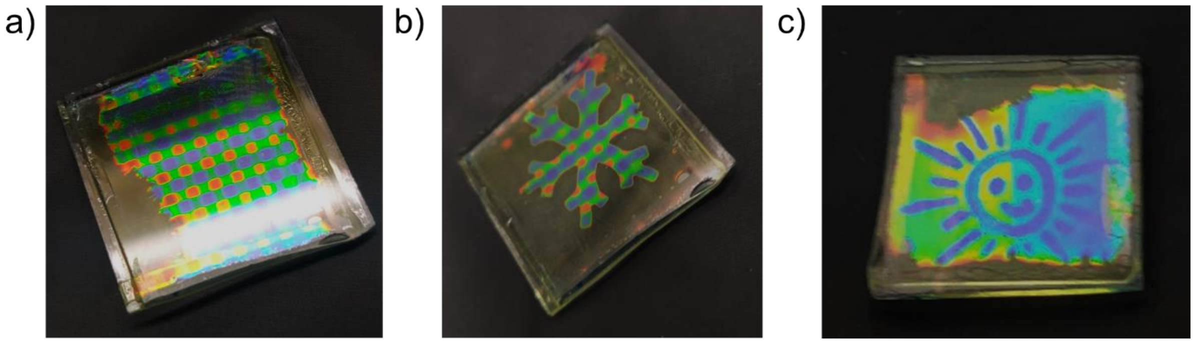

Supplementary Fig. S24. Images of the same sample (LC layer contacting $\boldsymbol{Z_S}$ 4.3 wt.%) with masked patterns generated by sequential irradiation. The pattern **a.** has been achieved by irradiating the sample with UV light till PSS followed by sequential irradiation with blue light through the lined mask which was rotated by 90 degree. The pattern **b.** was generated from the pattern **a.** by prolonged exposure to blue light though the snowflake mask (area around the snowflake was irradiated). The pattern **c.** was

generated in the similar manned as **a.** and **c.** by exposure to blue light through the hand-drawn pattern. In all cases colours originates from the reflecting by left-handed helical structure.

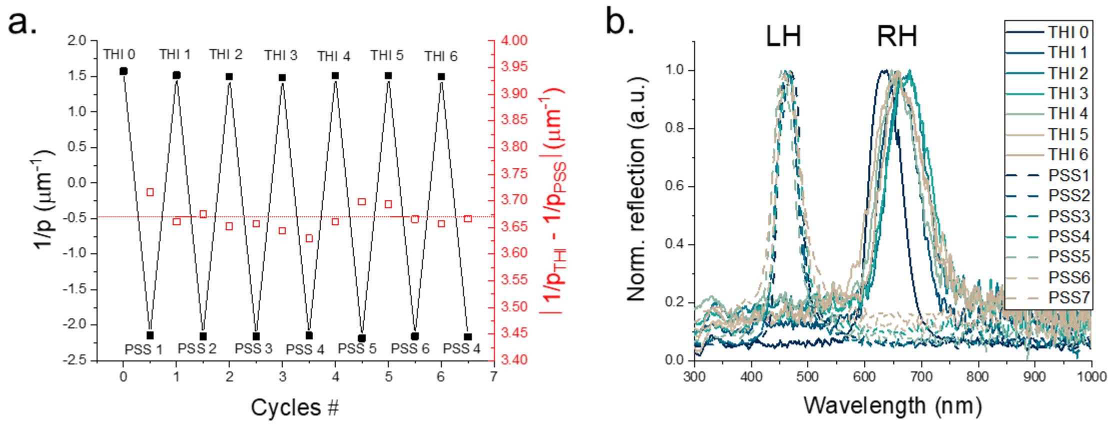

Supplementary Fig. S25. Performance of the materials (Z_S 4.3 wt.% in ZLI1083) upon cycling rotation of molecular motor. Each cycle consists of irradiation step with 365 nm UV light (PSS) and thermal step 110°C for 90 min (THI). A) Change in reversed pitch and dynamic range of the motor operation. Positive values correspond to the right-handed helical structure while the negative ones correspond to the left-handed structure. B) Corresponding reflection spectra upon cycling operation. RH and LH correspond to the right- and left-handed supramolecular helical structures.

Table S1. Helical twisting power ($HTP_{wt.\%}$ in μm^{-1}) of all 4 states of the motor **M1** in different liquid crystalline media. The positive values correspond to right-handed cholesteric helix, while the negative values – to the left-handed helix. The values were calculated based on wt.% concentration of motor in liquid crystal.

	(*R*,*P*)-$\boldsymbol{Z_S}$	(*R*,*M*)-$\boldsymbol{E_M}$*	(*R*,*P*)-$\boldsymbol{E_S}$#	(*R*,*M*)-$\boldsymbol{Z_M}$†	ΔHTP‡ (μm^{-1}) / ΔHTP (%)
MLC6816	+44.9	-29.8	+11.6	-61.9	106.8 / 238
5CB	+56.5	n.d.	+14.7	-77.2	133.7 / 237
E7	+57.9	-33.7	+16.6	-80.9	138.8 / 240
ZLI1083	+56.9	-35.3	+17.6	-83.3	140.2 / 246

*$\boldsymbol{E_M}$ were generated by irradiating $\boldsymbol{Z_S}$ with high intensity UV light (365 nm, 200 $mWcm^{-2}$) for 5s at 5°C. Value was corrected based on reaction conversion (81-86%) calculated from the ratio of the thermal equilibrated state. The value in 5CB cannot be determined due to crystallization of the matrix at 5°C.
#$\boldsymbol{E_S}$ were produced by sequential irradiation of DCM solution with 365 nm and 455 nm light
†$\boldsymbol{Z_M}$ Achieved by 365 nm irradiation of $\boldsymbol{Z_S}$ till photostationary state
‡Difference in HTP values of $\boldsymbol{Z_S}$ and $\boldsymbol{Z_M}$ which can be achieved by one step 365 nm exposure.

Crystals of Z_S-**M1** suitable for X-ray diffraction were obtained by solvent evaporation of CH_2Cl_2. A single crystal was mounted on a cryoloop and placed in the nitrogen stream (100 K) of a Bruker-AXS D8 Venture diffractometer, using Cu Kα radiation (λ = 1.54178 Å). The Bruker APEX4 software suite was used for data collection and processing, and a multi-scan absorption correction was applied, based on the intensities of symmetry-related reflections measured at different angular settings (SADABS-2016/2).[12] The structure was solved using SHELXT.[13] Subsequent refinement was done using SHELXL[14] in the OLEX2 software package.[15] Hydrogen atoms were generated by geometrical considerations and refined using a riding model.

Table S2 Crystal data and structure refinement for Z_S-M1

Empirical formula	$C_{50}H_{56}O_8$
Formula weight	784.94
Temperature/K	100.00
Crystal system	monoclinic
Space group	$P2_1/n$
a/Å	12.6086(5)
b/Å	22.4670(9)
c/Å	15.2100(6)
α/°	90
β/°	103.621(2)
γ/°	90
Volume/Å^3	4187.5(3)
Z	4
ρ_{calc}g/cm^3	1.245
μ/mm^{-1}	0.665
F(000)	1680.0
Crystal size/mm^3	0.27 × 0.23 × 0.075
Radiation	CuKα (λ = 1.54178)
2Θ range for data collection/°	7.158 to 136.488
Index ranges	$-15 \leq h \leq 15$, $-27 \leq k \leq 27$, $-18 \leq l \leq 18$
Reflections collected	162121
Independent reflections	7669 [R_{int} = 0.0889, R_{sigma} = 0.0310]
Data/restraints/parameters	7669/0/530
Goodness-of-fit on F^2	1.035
Final R indexes [I>=2σ (I)]	R_1 = 0.0420, wR_2 = 0.1081
Final R indexes [all data]	R_1 = 0.0491, wR_2 = 0.1143
Largest diff. peak/hole / e Å^{-3}	0.30/-0.28

Table S3. Optimized geometries of four **M1** states.

$\boldsymbol{E}_S$			
C	-1.06403	-0.46069	0.16032
C	-1.03585	0.27193	-1.03307
C	-2.42555	0.73051	-1.29716
C	-3.30896	-0.30249	-0.61145
C	-2.47949	-0.60069	0.65957
C	-2.79154	1.96686	-1.66413
C	-1.86511	3.15849	-1.86224
C	-2.48637	4.17829	-0.87766
C	-3.95532	3.84474	-0.94416
C	-4.141	2.56211	-1.47552
C	-5.03471	4.61535	-0.57563
C	-6.32785	4.11134	-0.7439
C	-6.48728	2.86122	-1.33991
C	-5.41824	2.06994	-1.74735
C	0.09601	-0.96628	0.70074
C	1.30839	-0.77048	0.0336
C	1.30304	-0.08524	-1.18245
C	0.14578	0.42728	-1.76005
C	-3.46812	-1.58648	-1.43311
C	-1.91375	3.72169	-3.28635
C	0.19879	1.02404	-3.13804
C	-5.67858	0.79766	-2.50277
C	2.51298	-1.34842	0.66325
C	-7.44632	4.95872	-0.28298
H	-4.28267	0.10985	-0.35503
H	-2.68247	-1.5897	1.07371
H	-2.68775	0.14325	1.43455
H	-0.84085	2.91861	-1.58386
H	-2.2772	5.21324	-1.1534
H	-2.10617	4.01074	0.1347
H	-4.9006	5.60629	-0.1537
H	0.08463	-1.52682	1.63018
H	-2.49457	-2.05471	-1.60107
H	-3.92054	-1.39891	-2.40615
H	-4.0963	-2.29964	-0.89436
H	-1.25019	4.58548	-3.37026
H	-2.92703	4.0505	-3.53161
H	-1.61194	2.98553	-4.03042
H	0.82588	0.41478	-3.79025
H	0.61525	2.03318	-3.13114
H	-0.80064	1.0655	-3.5648
H	-6.45426	0.95919	-3.25228
H	-6.02188	-0.00397	-1.84518
H	-4.7727	0.46713	-3.00536
O	3.63643	-1.35922	0.20615
H	2.31117	-1.80922	1.64775
O	-8.61599	4.64245	-0.22548
H	-7.127	5.9658	0.04301
O	-7.76811	2.39668	-1.61681
C	-8.40722	1.76189	-0.60435
O	-7.87063	1.53252	0.45357
O	2.5031	0.14722	-1.84233
C	3.04338	-0.88932	-2.53012
O	2.43446	-1.91227	-2.73387
C	4.41545	-0.60516	-2.99616
C	5.10813	0.53778	-2.61006

C	5.05104	-1.54529	-3.81325
C	6.4174	0.74948	-3.0182
C	6.34967	-1.34677	-4.22879
C	7.04543	-0.19869	-3.8279
H	4.6297	1.26975	-1.97275
H	4.5151	-2.43713	-4.11389
H	6.93428	1.64263	-2.69746
H	6.85285	-2.06985	-4.85947
C	-9.79228	1.38541	-0.95119
C	-10.34855	1.64297	-2.19992
C	-10.56908	0.75345	0.02529
C	-11.65669	1.27691	-2.48623
C	-11.87002	0.39018	-0.24277
C	-12.42296	0.6466	-1.50422
H	-9.75963	2.13476	-2.96321
H	-10.13865	0.55336	0.99863
H	-12.0641	1.48698	-3.46492
H	-12.48181	-0.09707	0.5069
O	-13.70125	0.24938	-1.67502
C	-14.31496	0.43873	-2.95729
H	-13.72521	-0.08158	-3.71803
H	-14.32934	1.50616	-3.19706
C	-15.71706	-0.12264	-2.87391
H	-16.30031	0.40539	-2.1171
H	-15.69286	-1.18458	-2.62213
H	-16.21522	-0.00695	-3.8384
O	8.3168	-0.09584	-4.26915
C	9.09347	1.03765	-3.85682
H	9.13118	1.06827	-2.76391
H	8.61293	1.95295	-4.21482
C	10.47811	0.88042	-4.44452
H	10.43483	0.84418	-5.53474
H	10.95067	-0.0335	-4.07954
H	11.09671	1.7309	-4.15159
$\boldsymbol{E}_{\mathrm{M}}$			
C	3.10045	0.10772	4.77773
C	2.81537	0.42537	3.44281
C	3.98007	1.11327	2.84789
C	5.17403	0.76011	3.74305
C	4.48762	0.56325	5.12107
C	3.94662	2.02736	1.85868
C	2.77691	2.94528	1.48263
C	3.51168	4.26634	1.13234
C	4.8561	3.81717	0.64256
C	5.09991	2.50389	1.06755
C	5.76762	4.49681	-0.12938
C	6.94261	3.84529	-0.51703
C	7.12493	2.51272	-0.15719
C	6.21128	1.79114	0.60802
C	2.18399	-0.54776	5.56429
C	0.96056	-0.92651	5.00259
C	0.73243	-0.67861	3.65181
C	1.65013	-0.03542	2.82369
C	6.28575	1.7972	3.86481
C	1.72163	3.20443	2.55338
C	1.38803	0.07058	1.34794
C	6.41604	0.32018	0.83644
C	-0.05533	-1.60643	5.83452
C	7.95358	4.56256	-1.3236

H	5.5925	-0.20679	3.4437
H	5.02361	-0.14284	5.75657
H	4.45065	1.52367	5.6464
H	2.30033	2.58242	0.56564
H	2.96825	4.86444	0.39987
H	3.62248	4.86928	2.04002
H	5.59798	5.51833	-0.44837
H	2.38548	-0.78189	6.60275
H	6.92251	1.53596	4.71395
H	5.85872	2.78531	4.05514
H	6.92343	1.86911	2.98801
H	1.1107	4.05922	2.25224
H	1.05099	2.36636	2.72182
H	2.2012	3.45187	3.50402
H	0.69574	0.88219	1.11316
H	2.31725	0.25068	0.81083
H	0.94931	-0.85228	0.96888
H	6.78318	-0.16121	-0.06972
H	7.14446	0.12727	1.62724
H	5.47738	-0.15358	1.11677
O	7.81517	5.7075	-1.70456
O	0.11238	-1.8762	7.00674
H	8.87292	4.0084	-1.5687
H	-1.00613	-1.86179	5.34161
O	8.3045	1.87523	-0.54394
C	8.30434	1.22717	-1.73775
O	7.31895	1.15789	-2.43249
O	-0.49959	-1.04104	3.10556
C	-0.61174	-2.27868	2.55875
O	0.31447	-3.05234	2.51683
C	9.62419	0.64776	-2.05672
C	9.73861	-0.18325	-3.17618
C	10.75584	0.92062	-1.29383
C	10.95573	-0.72948	-3.51892
C	11.98961	0.38514	-1.63331
C	12.09285	-0.44597	-2.75051
H	8.85989	-0.39702	-3.77223
H	10.68209	1.56516	-0.42772
H	11.05572	-1.37835	-4.3807
H	12.85297	0.6199	-1.02733
C	-1.97122	-2.53813	2.04495
C	-2.19063	-3.69333	1.28725
C	-3.03799	-1.68345	2.3058
C	-3.44656	-3.98121	0.80059
C	-4.31059	-1.96775	1.83246
C	-4.5187	-3.12129	1.07412
H	-1.36229	-4.36016	1.08152
H	-2.88245	-0.78715	2.892
H	-3.62718	-4.86975	0.20762
H	-5.12255	-1.29114	2.05744
O	13.24154	-1.01846	-3.16535
C	14.44543	-0.73829	-2.43643
H	14.32238	-1.06397	-1.39927
H	14.6258	0.34074	-2.44429
C	15.57342	-1.4844	-3.11279
H	15.68501	-1.16015	-4.14916
H	15.38974	-2.56039	-3.09767
H	16.50842	-1.28515	-2.58558
O	-5.71249	-3.48844	0.56552
C	-6.85379	-2.66108	0.8343

H	-6.67543	-1.66001	0.43049
H	-6.99198	-2.58065	1.91651
C	-8.05132	-3.31087	0.17847
H	-8.21712	-4.31125	0.58275
H	-7.90816	-3.38624	-0.90114
H	-8.94213	-2.70911	0.36869
***Z*S**			
C	-0.43555	-0.48229	5.64634
C	0.1054	-0.18807	4.38385
C	-0.29801	1.18222	4.0199
C	-1.56882	1.45517	4.813
C	-1.29475	0.66544	6.11784
C	0.34257	2.09653	3.28116
C	-0.29526	3.38337	2.77708
C	0.25104	3.44501	1.32881
C	1.60704	2.79455	1.45387
C	1.67195	2.04249	2.63856
C	2.69528	2.89231	0.62077
C	3.89336	2.27221	0.99589
C	3.9626	1.62704	2.22642
C	2.87623	1.50669	3.09254
C	-0.18027	-1.68686	6.25688
C	0.62353	-2.62511	5.59607
C	1.07687	-2.33888	4.31352
C	0.78922	-1.14956	3.64469
C	-2.81439	0.9295	4.09657
C	0.14428	4.60187	3.59142
C	1.15541	-1.00237	2.19504
C	3.07504	0.9058	4.45426
C	0.95219	-3.91212	6.24261
C	5.0718	2.33114	0.10545
H	-1.6848	2.52004	5.01467
H	-2.20957	0.3287	6.60859
H	-0.74111	1.28742	6.8279
H	-1.38265	3.31158	2.78472
H	-0.39014	2.86543	0.65747
H	0.31167	4.46364	0.9419
H	2.65579	3.44874	-0.30825
H	-0.58196	-1.92952	7.23361
H	-2.72507	-0.14309	3.90306
H	-2.95717	1.43613	3.13956
H	-3.70447	1.09183	4.70908
H	1.23328	4.7008	3.58351
H	-0.18127	4.51427	4.63028
H	-0.28321	5.51568	3.17201
H	0.88309	-1.90781	1.64923
H	2.22496	-0.84419	2.05648
H	0.61962	-0.1679	1.74854
H	4.00725	1.27283	4.88684
H	3.13805	-0.18321	4.41147
H	2.25413	1.17092	5.11675
O	0.52221	-4.24695	7.3281
O	5.05786	2.85763	-0.98923
H	1.6313	-4.57846	5.68874
H	5.99278	1.85899	0.48033
O	1.92638	-3.22819	3.65842
C	1.38003	-4.20399	2.88821
O	0.20603	-4.48216	2.9377
O	5.20285	1.15231	2.65439

C	5.41872	-0.18593	2.57812
O	4.6135	-0.94117	2.0885
C	2.38465	-4.83445	2.00951
C	3.68061	-4.33659	1.90651
C	2.00499	-5.92317	1.21716
C	4.59229	-4.89952	1.02599
C	2.90136	-6.49281	0.33977
C	4.20063	-5.97773	0.23054
H	3.98345	-3.48612	2.5019
H	0.99696	-6.31236	1.29266
H	5.58908	-4.48639	0.96189
H	2.61877	-7.3334	-0.2824
C	6.72543	-0.57896	3.13986
C	7.60852	0.34535	3.70853
C	7.08576	-1.9221	3.09041
C	8.82152	-0.0729	4.21208
C	8.30178	-2.35657	3.59369
C	9.17793	-1.42661	4.1582
H	7.34236	1.39319	3.75589
H	6.40304	-2.63738	2.64932
H	9.51493	0.6322	4.65431
H	8.55532	-3.40561	3.5407
O	5.00015	-6.58147	-0.67247
C	6.33061	-6.07351	-0.85197
H	6.88003	-6.16585	0.08973
H	6.27822	-5.0145	-1.12186
C	6.982	-6.8829	-1.95063
H	6.42837	-6.77935	-2.88574
H	7.02331	-7.93992	-1.6809
H	8.00129	-6.52626	-2.11102
O	10.38419	-1.73832	4.67416
C	10.79962	-3.11167	4.66111
H	10.83079	-3.46811	3.62735
H	10.07169	-3.71173	5.21506
C	12.16744	-3.18064	5.30295
H	12.13034	-2.82293	6.33362
H	12.88645	-2.57688	4.74597
H	12.51492	-4.21556	5.30849
***Z*M**			
C	1.22796	0.12357	5.40574
C	0.82334	0.43826	4.10525
C	1.45484	1.70391	3.69274
C	2.63098	1.93832	4.64301
C	2.20399	1.14719	5.90974
C	1.01006	2.55195	2.74506
C	1.86498	3.51365	1.91714
C	1.0869	3.5859	0.57457
C	-0.32067	3.20493	0.93112
C	-0.36012	2.63104	2.20581
C	-1.47073	3.35874	0.18861
C	-2.69453	2.95449	0.7299
C	-2.72247	2.49355	2.04548
C	-1.5846	2.35678	2.83249
C	0.73909	-0.99383	6.04529
C	-0.1559	-1.83337	5.3752
C	-0.46287	-1.55316	4.04239
C	0.05071	-0.45886	3.3549
C	2.89218	3.39794	5.00666
C	3.29724	3.04952	1.66176

C	-0.19165	-0.35445	1.87465
C	-1.74797	2.01181	4.28676
C	-0.71081	-2.96787	6.13934
C	-3.88782	3.05775	-0.13431
H	3.5404	1.47911	4.24082
H	3.05149	0.69868	6.42991
H	1.70881	1.82583	6.61218
H	1.86265	4.51098	2.36996
H	1.50638	2.85863	-0.12851
H	1.14993	4.56862	0.10512
H	-1.44334	3.77785	-0.81217
H	1.02548	-1.22871	7.06549
H	1.97022	3.85843	5.37094
H	3.63409	3.44799	5.80772
H	3.26098	3.99643	4.17682
H	3.28763	2.03883	1.24543
H	3.77177	3.71069	0.93209
H	3.92065	3.04002	2.55285
H	0.53927	0.30624	1.41315
H	-0.10176	-1.3415	1.41876
H	-1.18972	0.02263	1.64841
H	-2.60392	2.55011	4.69503
H	-1.92829	0.94565	4.43675
H	-0.85965	2.28521	4.85159
O	-1.51019	-3.7893	5.74124
O	-4.99155	2.61408	0.10433
H	-3.69477	3.58354	-1.08757
H	-0.33176	-3.02125	7.1764
O	-1.39142	-2.32627	3.36306
C	-1.05047	-3.5702	2.95518
O	0.06778	-4.0112	3.06849
O	-3.95087	2.23258	2.64316
C	-4.40516	0.95962	2.57396
O	-3.77734	0.07458	2.04149
C	-2.20144	-4.27527	2.35318
C	-3.41767	-3.63709	2.12783
C	-2.06341	-5.62364	2.01205
C	-4.49154	-4.32359	1.57952
C	-3.12362	-6.31896	1.47203
C	-4.34839	-5.67401	1.25686
H	-3.53334	-2.58962	2.37363
H	-1.11739	-6.12246	2.184
H	-5.42395	-3.80269	1.41305
H	-3.03206	-7.36687	1.21275
C	-5.71652	0.79564	3.23325
C	-6.4728	1.88718	3.67179
C	-6.21049	-0.4914	3.41936
C	-7.69484	1.68779	4.27778
C	-7.43402	-0.70715	4.03502
C	-8.18498	0.38918	4.46413
H	-6.10336	2.89426	3.52937
H	-5.62514	-1.33879	3.0843
H	-8.29341	2.52529	4.61542
H	-7.78826	-1.71873	4.17328
O	-5.33139	-6.43758	0.73488
C	-6.6224	-5.84754	0.53081
H	-6.53165	-5.01483	-0.17297
H	-6.99521	-5.4593	1.48339
C	-7.53198	-6.92685	-0.01246
H	-7.61161	-7.75539	0.69395

H	-7.1535	-7.30927	-0.96233
H	-8.5297	-6.5157	-0.17759
O	-9.38993	0.2956	5.06462
C	-9.95461	-1.00812	5.26322
H	-9.28391	-1.5981	5.89505
H	-10.05183	-1.50862	4.29527
C	-11.30467	-0.82517	5.91977
H	-11.96557	-0.23278	5.2841
H	-11.20185	-0.32487	6.88456
H	-11.76543	-1.80124	6.08361

Table S4. Representative photoresponsive LC chiral dopants examples in recent 5 years.

Switch	LC Hosts	β [µm] Initial	β [µm] PSS	Number of Helix Inversions	Reference
**Triptycene–hydrazone**	5CB	+53	+107 (340 nm)	0	*Nat. Chem.* 2024, 16, 2084–2090.
**Isosorbide-based Hydrazone**	5CB	+35	+57 (340 nm)	0	*J. Am. Chem. Soc.* 2024, 146, 35, 24561–24569
**Isosorbide-based Hydrazone**	5CB	+21	-13	2 (reversible)	*Chem. Sci.*, 2024, 15, 17041
**DAE**	LCM17	144.6	57.3	0	*Nat. Photon.* 2022, 16, 226–234.
**DAE**	ZLI1083	-242.8	+203.9	2 (reversible)	*Adv. Funct. Mater*. 2024, 34, 2312831
**Binaphthol-based Hydrazone**	5CB	-31 （PSS_{340}）	+23 （455 nm）	2 (reversible)	*CCS Chem*. 2024, 6, 2011–2020
**Azobenzene**	5CB; E7; ZLI1132	-13 (E7)	+45 (530 nm)	2 (reversible)	*Angew. Chem. Int. Ed.* 2023, 62, e202216600
**Typical I-gen motor**	ZLI1083	+76	-89 (300 nm)	2 (reversible)	*Adv. Mater.* 2020, 32, 2004420
**CHO-motor**	**5CB; E7; ZLI1083; MLC6816**	**+57 (ZLI1083)**	**-83 (365nm)**	**4 (sequential)**	***This work***

12. References

1. Cano, R. *Bull. Soc. Franc. Minéral. Cristallogr.*, **91**, 20 – 27 (1968).
2. Gerber, P. R. On the determination of the cholesteric screw sense by the Grandjean–Cano-method. *Z. Nat.* **35**, 619–622 (1980).
3. Li, Z. & Zhang, Y. An Efficient and User-Friendly Method for the Synthesis of Hexagonal-Phase $NaYF_4$:Yb,Er/Tm Nanocrystals with Controllable Shape and Upconversion Fluorescence. *Nanotechnology* **19**, 345606-345606 (2008).
4. Qian, H. S. & Zhang, Y. Synthesis of hexagonal-phase core-shell $NaYF_4$ nanocrystals with tunable upconversion fluorescence. *Langmuir* **24**, 12123-12125 (2008).
5. Sheng, J., Danowski, W., Sardjan, A.S. et al. Formylation boosts the performance of light-driven overcrowded alkene-derived rotary molecular motors. *Nat. Chem*. **16**, 1330–1338 (2024).
6. Najibi, A.; Goerigk, L. *J. Comput. Chem.*, **41**, 2562 (2020).
7. V. Barone, M. Cossi, *J. Phys. Chem. A* **102**, 1995–2001 (1998).
8. F. Neese, F. Wennmohs, U. Becker, C. Riplinger, *J. Chem. Phys.* **152**, 224108 (2020).
9. Stranius, K. & Börjesson, K. Determining the Photoisomerization Quantum Yield of Photoswitchable Molecules in Solution and in the Solid State. *Sci. Rep.* **7**, 41145 (2017).
10. Kuhn, H. J., Braslavsky, S. E. & Schmidt, R. Chemical actinometry (IUPAC Technical Report). *Pure Appl. Chem.* **76**, 2105–2146 (2004).
11. Hoops, S. *et al.* COPASI--a COmplex PAthway SImulator. *Bioinformatics* **22**, 3067–3074 (2006).
12. Krause, L.; Herbst-Irmer, R.; Sheldrick, G. M.; Stalke, D., Comparison of silver and molybdenum microfocus X-ray sources for single-crystal structure determination. *J. Appl. Cryst.* **48** (1), 3-10 (2015).
13. Sheldrick, G. M., SHELXT–Integrated space-group and crystal-structure determination. *Acta Cryst. A* **71** (1), 3-8 (2015).
14. Sheldrick, G. M., A short history of SHELX. *Acta Cryst. A* **64** (1), 112-122 (2008).
15. Dolomanov, O. V.; Bourhis, L. J.; Gildea, R. J.; Howard, J. A.; Puschmann, H., OLEX2: a complete structure solution, refinement and analysis program. *J. Appl. Cryst.* **42** (2), 339-341 (2009).
